# Digital Twins of Acute Hypoxemic Respiratory Failure Patients Suggest a Mechanistic Basis for Success and Failure of Noninvasive Ventilation

**DOI:** 10.1097/CCM.0000000000006337

**Published:** 2024-05-29

**Authors:** Liam Weaver, Hossein Shamohammadi, Sina Saffaran, Roberto Tonelli, Marianna Laviola, John G. Laffey, Luigi Camporota, Timothy E. Scott, Jonathan G. Hardman, Enrico Clini, Declan G. Bates

**Affiliations:** 1 School of Engineering, University of Warwick, Coventry, United Kingdom.; 2 Respiratory Diseases Unit, Department of Medical and Surgical Sciences, University Hospital of Modena, University of Modena and Reggio Emilia, Modena, Italy.; 3 Anaesthesia and Critical Care, Injury Inflammation and Recovery Sciences, School of Medicine, University of Nottingham, Nottingham, United Kingdom.; 4 Anaesthesia and Intensive Care Medicine, Galway University Hospitals and School of Medicine, University of Galway, Galway, Ireland.; 5 Intensive Care Medicine, Guy’s and St Thomas’ NHS Foundation Trust and Division of Asthma Allergy and Lung Biology, King’s College London, London, United Kingdom.; 6 Academic Department of Military Anaesthesia and Critical Care, Royal Centre for Defence Medicine, ICT Centre, Birmingham, United Kingdom.; 7 Nottingham University Hospitals NHS Trust, Nottingham, United Kingdom.

**Keywords:** acute hypoxemic respiratory failure, digital twins, noninvasive ventilation

## Abstract

**OBJECTIVES:**

To clarify the mechanistic basis for the success or failure of noninvasive ventilation (NIV) in acute hypoxemic respiratory failure (AHRF).

**DESIGN:**

We created digital twins based on mechanistic computational models of individual patients with AHRF.

**SETTING:**

Interdisciplinary Collaboration in Systems Medicine Research Network.

**SUBJECTS:**

We used individual patient data from 30 moderate-to-severe AHRF patients who had failed high-flow nasal cannula (HFNC) therapy and subsequently underwent a trial of NIV.

**INTERVENTIONS:**

Using the digital twins, we evaluated lung mechanics, quantified the separate contributions of external support and patient respiratory effort to lung injury indices, and investigated their relative impact on NIV success or failure.

**MEASUREMENTS AND MAIN RESULTS:**

In digital twins of patients who successfully completed/failed NIV, after 2 hours of the trial the mean (sd) of the change in total lung stress was –10.9 (6.2)/–0.35 (3.38) cm H_2_O, mechanical power –13.4 (12.2)/–1.0 (5.4) J/min, and total lung strain 0.02 (0.24)/0.16 (0.30). In the digital twins, positive end-expiratory pressure (PEEP) produced by HFNC was similar to that set during NIV. In digital twins of patients who failed NIV vs. those who succeeded, intrinsic PEEP was 3.5 (0.6) vs. 2.3 (0.8) cm H_2_O, inspiratory pressure support was 8.3 (5.9) vs. 22.3 (7.2) cm H_2_O, and tidal volume was 10.9 (1.2) vs. 9.4 (1.8) mL/kg. In digital twins, successful NIV increased respiratory system compliance +25.0 (16.4) mL/cm H_2_O, lowered inspiratory muscle pressure –9.7 (9.6) cm H_2_O, and reduced the contribution of patient spontaneous breathing to total driving pressure by 57.0%.

**CONCLUSIONS:**

In digital twins of AHRF patients, successful NIV improved lung mechanics, lowering respiratory effort and indices associated with lung injury. NIV failed in patients for whom only low levels of positive inspiratory pressure support could be applied without risking patient self-inflicted lung injury due to excessive tidal volumes.

**KEY POINTS**

**Question:** We aimed to clarify the mechanistic basis for success and failure of noninvasive ventilation.

**Findings:** In digital twins of patients who succeeded on noninvasive ventilation (NIV), the positive inspiratory pressure provided by NIV improved lung mechanics, leading to reduced respiratory effort and reductions in lung stress, strain, driving pressure, and mechanical power. NIV failed in patients for whom only low levels of positive inspiratory pressure support could be applied without increasing the risk of patient self-inflicted lung injury due to excessive tidal volumes.

**Meaning:** Reduction of patients’ respiratory efforts by NIV predicts success because it reduces the risk of lung injury.

Noninvasive ventilation (NIV) is increasingly used to support spontaneous breathing in patients with de novo acute hypoxemic respiratory failure (AHRF) (1). When successful, NIV may reduce risk of intubation, death, and length of ICU stay (1). However, NIV failure is associated with increased mortality in patients who are subsequently intubated (1–3). In particular, significant uncertainty exists regarding the potential for additional lung injury if high respiratory effort is not controlled by NIV (3). A recent expert panel of the European Society of Intensive Care Medicine reported insufficient evidence to make a recommendation for, or against, the use of NIV compared with conventional oxygen therapy and suggested that future research should focus on the potential role of high vs. low respiratory drive in determining patient suitability and likelihood of success (4).

Much of this uncertainty may be attributed to the significantly greater difficulties associated with measuring many key patient parameters and, in particular, indices associated with lung injury in spontaneously breathing patients. While respiratory rate (RR) is measurable and commonly available, measuring tidal volume (Vt) requires specialized equipment and signal processing, for example, by numerical integration of respiratory flow measured by a pneumotachograph. This also introduces the potential for error, due to some portion of the flow directed to the patient being included in the measurement. To assess inspiratory effort directly, measurement of tidal changes in esophageal pressure (ΔPes) and dynamic transpulmonary pressure require the placement of a dedicated esophageal pressure transducer, while many other key indices of potential lung injury such as mechanical power, lung stress, lung strain, and compliance are difficult or impossible to measure accurately in patients receiving noninvasive respiratory support.

A potential solution to these challenges is to create digital twins (5), based on mechanistic, computational models calibrated using the complete set of data measured in each individual patient. Such digital twins can then be used to estimate nonmeasurable parameters of interest and perform additional experiments that would not be possible in vivo, enriching the data generated by clinical trials and assisting in interpreting and explaining their findings.

Here, we demonstrate the first application of this approach in the context of AHRF, by creating a cohort of digital twins based on detailed individual patient data from 30 moderate-to-severe AHRF patients reported in (6). In that study, the magnitude of the change in inspiratory effort within the first 2 hours of NIV, as measured by reduction in ΔPes, was found to be an accurate predictor of NIV outcome at 24 hours. However, the mechanistic basis for the statistical association between ΔPes reduction and outcome remains unclear, since many important indices associated with lung injury could not be measured in vivo during the trial.

## METHODS

In the following, we summarize the patient data that were extracted from measurements made in a cohort of AHRF patients and describe how these data were used to develop the digital twins employed in this study. We also detail how various ventilator-induced lung injury indices were calculated and how risk thresholds for these indices were defined. For full methodological details, the interested reader is referred to the **Supplementary Material** (http://links.lww.com/CCM/H553).

### Ethical Approval

Ethical approval was not required as no patients were involved in the study, and patient data on which the digital twins were based were previously published in (6).

### Study Population

Anonymized individual patient data were taken from a prospective observational cohort study of 30 patients, performed in a single eight-bed respiratory ICU at the University Hospital of Modena, Italy, between February and October of 2019 (6). Inclusion criteria were age older than 18 years; the presence of de novo AHRF with a Pao_2_/Fio_2_ ratio less than 200 mm Hg on high-flow nasal cannula (HFNC) therapy with the flow set at 60 L/min; and the candidate’s approval for receiving a NIV trial by the attending ICU staff, whose decisions were made based on clinical conditions, being blinded to the purpose of the study. In the NIV trial, positive end-expiratory pressure (PEEP) was initially set at 6 cm H_2_O and subsequently titrated (4–8 cm H_2_O) to target a transcutaneous oxyhemoglobin saturation (Spo_2_) greater than 92% with a delivered Fio_2_ less than 70%. Inspiratory pressure support (PS) was initially set at 10 cm H_2_O and then progressively modified to target an expiratory Vt of predicted body weight less than 9.5 mL/kg and a RR less than 30 breaths/min. Fio_2_ was increased to target a Spo_2_ of 88–94%. NIV failure (*n* = 12/30) was defined by the need for endotracheal intubation or by death.

### Creation of a Cohort of Digital Twins

A complete set of the available physiologic measurements were extracted from the study data for each patient while on HFNC therapy (i.e., before the NIV trial) and at 2 hours after initiation of NIV. These data were used to further develop a previously validated cardiopulmonary simulator (7–11) (**Figs. S1** and **S2**, http://links.lww.com/CCM/H553) to represent spontaneously breathing AHRF patients receiving noninvasive respiratory support. Data from five of the 30 patients were excluded due to concerns about unrealistically large Vt measurements, presumed to be due to some portion of the flow directed to the patient being erroneously included in the integrated flow signal. Advanced global optimization algorithms running on high performance computing facilities were used to calibrate the parameters of the simulator for each individual patient so that its outputs matched as closely as possible the corresponding data (**Tables S2** and **S3**, http://links.lww.com/CCM/H553); for full details and a complete description of the simulator, see the Supplementary Material (http://links.lww.com/CCM/H553).

### Risk Thresholds for Indices of Lung Injury

Although the existence of any safe limits is still debated for some lung injury indices (12), as an aid to visualization when comparing the data before and after initiation of NIV, we defined some potential risk thresholds for the various indices based on the latest results available in the literature (12–15). These are summarized in **Table S1** (http://links.lww.com/CCM/H553).

### Calculation of Driving Pressure in Spontaneous Breathing With Pressure Support

Recent studies have shown that a brief inspiratory hold technique can provide satisfactory estimation of driving pressure during PS ventilation with spontaneous effort (16). We are able to analyze the distending inspiratory pressure within each digital twin, giving a direct measure of driving pressure in these patients (**Fig. S3**, http://links.lww.com/CCM/H553).

### Separating the Contributions of Patient Effort and External Respiratory Support to Lung Injury Indices

To separate the two, the digital twins had their external respiratory support removed, while their spontaneous respiratory efforts were kept unchanged, and the values of all lung injury indices were recalculated. The differences between the original lung injury indices and those produced when only spontaneous respiratory efforts were present were then attributed to the external respiratory support.

### Statistical Analysis

Data are presented as mean (sd) or shown graphically with box-whisker plots using median, interquartile, and total ranges. To avoid violation of underlying distribution assumptions, variables were compared using the Wilcoxon rank-sum test. A two-sided *p* value of less than 0.05 was considered significant. Results that did not achieve statistical significance are denoted in the text with an *.

## RESULTS

### Digital Twins Accurately Reproduce All Measurements Made in Original Patients

**Figure 1.**
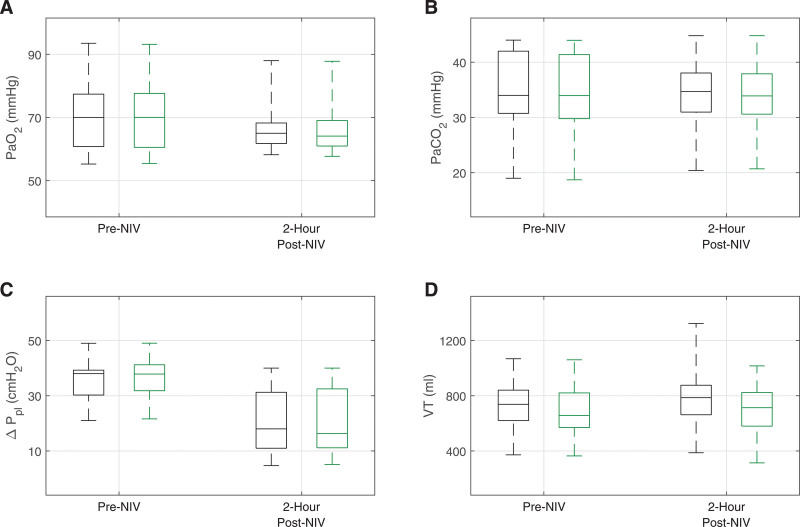
Patient data distributions (*black*) compared with digital twin outputs (*green*) while on high-flow nasal cannula therapy (before initiation of noninvasive ventilation [NIV]) and 2 hr after initiation of NIV, median, interquartile ranges, and actual ranges for PaO_2_ (**A**), PaCO_2_ (**B**), tidal change in pleural pressure (ΔP_pl_) (**C**), and tidal volume (Vt) (**D**).

A comparison of the digital twins’ outputs for Pao_2_, Paco_2_, tidal change in pleural pressure (ΔP_pl_), and Vt vs. the individual patient measurements made before the NIV trial (i.e., while on HFNC therapy) and after 2 hours of NIV is shown in **Figure 1** (for data for each individual patient, see **Tables S4–S6**, http://links.lww.com/CCM/H553). In the case of ΔP_pl_, we are comparing the actual ΔP_pl_ (available in the digital twins) with its surrogate, ΔPes (as measured in patients by esophageal manometry). Mean absolute percentage error/mean absolute bias between the data and the digital twins’ outputs under HFNC across the cohort were 0.44%/0.07 mm Hg for Pao_2_, 1.07%/0.33 mm Hg for Paco_2_, 3.2%/–0.9 cm H_2_O for ΔP_pl_, and 6.35%/40.77 mL for Vt. After 2 hours of the NIV trial, the errors were 1.21%/0.36 mm Hg for Pao_2_, 1.14%/0.26 mm Hg for Paco_2_, 1.97%/–0.36 cm H_2_O for ΔP_pl_, and 10.02%/87.29 mL for Vt.

### Digital Twins Reveal Effects of NIV on Lung Injury Indices

**Figure 2.**
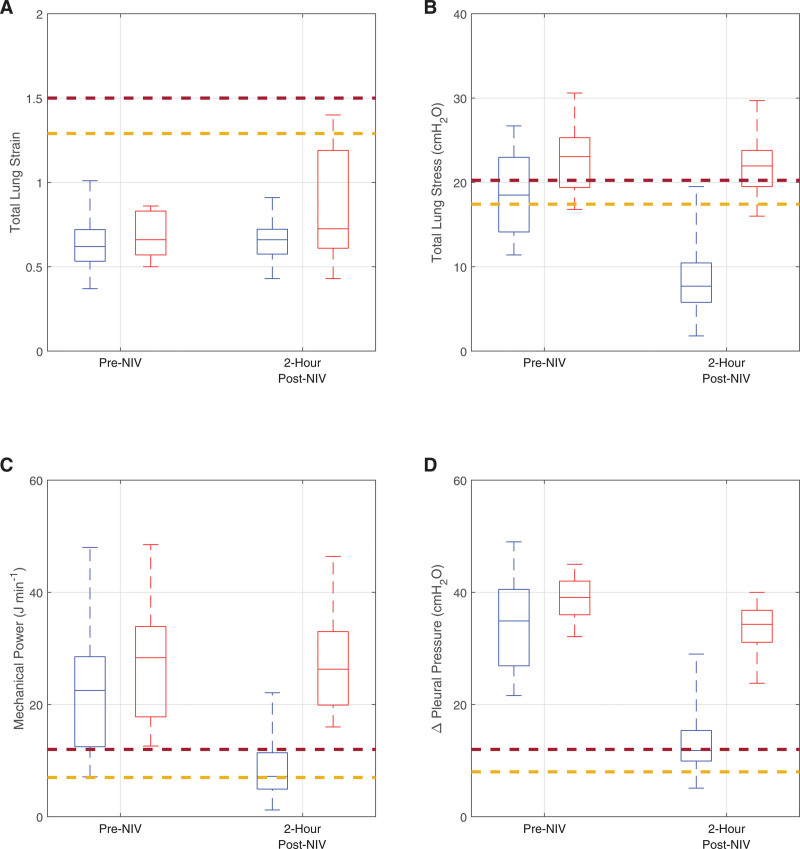
Lung injury indices on HFNC (pre-noninvasive ventilation [NIV]) and after 2 hr of the NIV trial, median, interquartile ranges, and actual ranges; total lung strain (**A**), total lung stress (**B**), mechnical power (**C**), and tidal change in pleural pressure (**D**). *Blue* (*red*)—digital twins of patients in which NIV succeeded (failed), *red dash*—higher risk threshold, and *yellow dash*—lower risk threshold.

**Figure 3.**
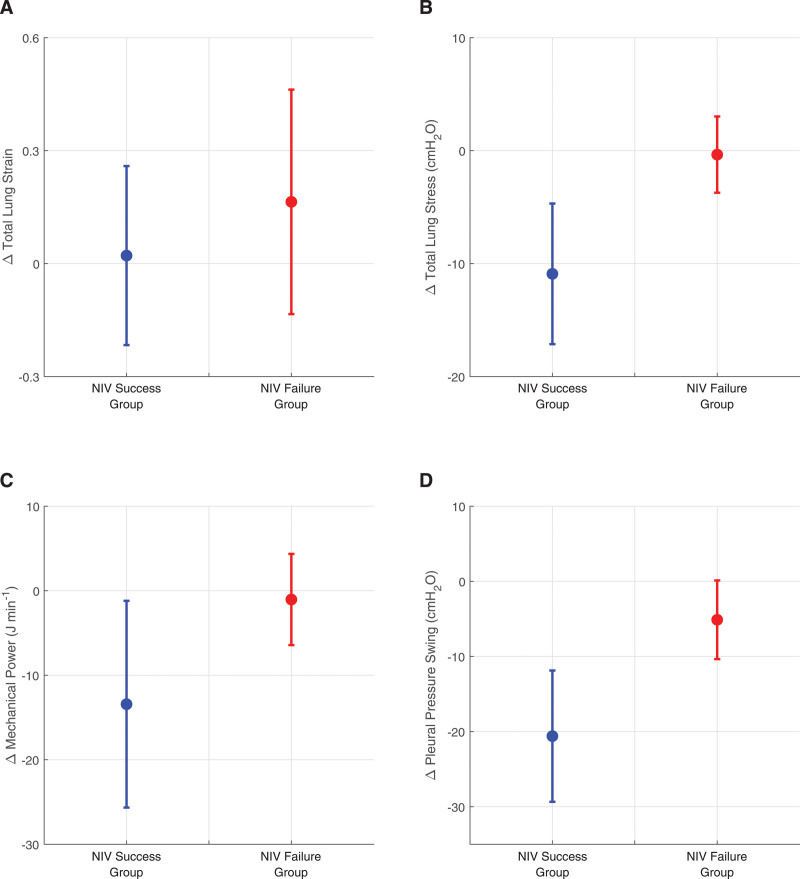
Changes in lung injury indices after 2 hr of the noninvasive ventilation (NIV) trial in digital twins of the success and failure patient subgroups; change in total lung strain (**A**), change in total lung stress (**B**), change in mechanical power (**C**), and change in tidal pleural pressure swing (**D**). *Circle*—mean value; *whisker*—sd. Change in total lung strain was not statistically significant.

**Figure 2** shows the values of total lung strain, total lung stress, mechanical power, and ΔP_pl_ calculated in the digital twins of the NIV success and failure groups after two hours of the NIV trial, compared with the values calculated in the digital twins of the same patients before the trial, that is, while receiving HFNC therapy (for values for individual patients, **Tables S7–S14**, http://links.lww.com/CCM/H553). **Figure 3** shows the corresponding changes in the values of the lung injury indices. In the digital twins of patients in whom NIV was successful vs. those who failed NIV, the change in mean total lung strain was +0.02 vs. +0.16*, the change in mean total lung stress was –10.9 vs. –0.35 cm H_2_O, the change in mean mechanical power was –13.4 vs. –1.0 J/min, and the change in mean ΔP_pl_ was –20.6 vs. –5.1 cm H_2_O.

### Mechanistic Basis for Reduced Inspiratory Effort in Successful NIV Patients

**Figure 4.**
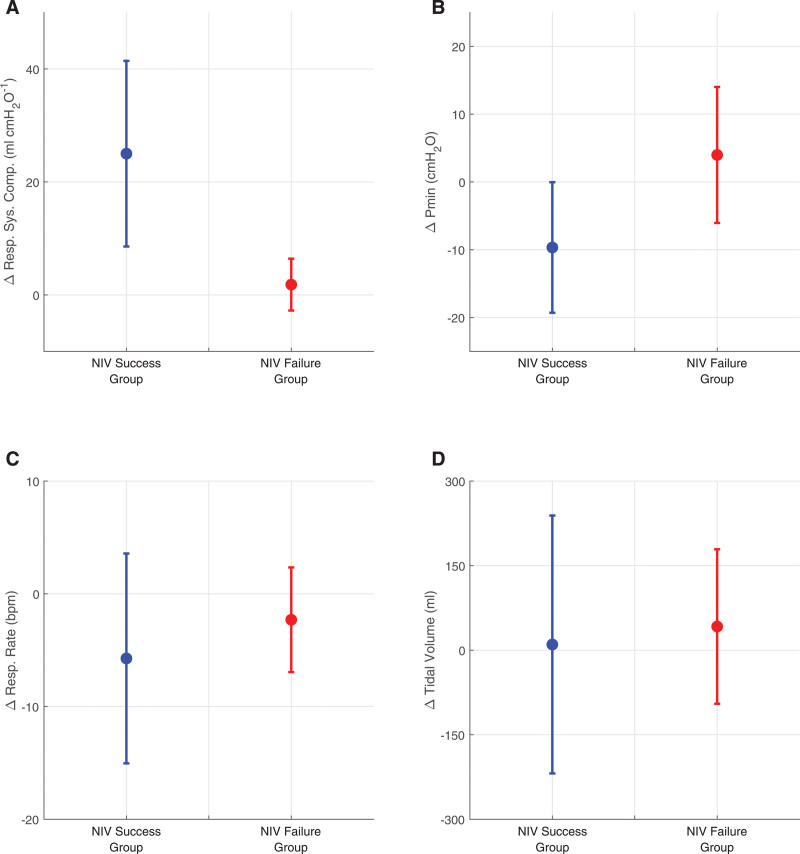
Changes in lung mechanics after 2 hr of the noninvasive ventilation (NIV) trial in digital twins of the success and failure patient subgroups; change in respiratory system compliance (**A**), change in muscular pressure (Pmin) (**B**), change in respiratory rate (**C**), and change in tidal volume (**D**). *Circle*—mean value; *whisker*—sd.

Analysis of the data provided by the digital twins suggests that, in patients for whom it was successful, NIV delivered positive inspiratory pressures that were sufficient to produce alveolar recruitment, with mean respiratory system compliance increasing by an average of 25.0 mL/cm H_2_O within 2 hours (**Fig. 4*A***). In contrast, mean respiratory system compliance was effectively unchanged (+1.83 mL/cm H_2_O) in the digital twins of patients that failed NIV. Improvements in lung compliance allowed for a mean reduction in inspiratory muscle pressure of 9.7 cm H_2_O in the NIV success group, whereas respiratory effort was unchanged or increased in the NIV failure group (**Fig. 4**).

### Contributions of Patient Effort and External Respiratory Support to Lung Injury Indices

**Figure 5.**
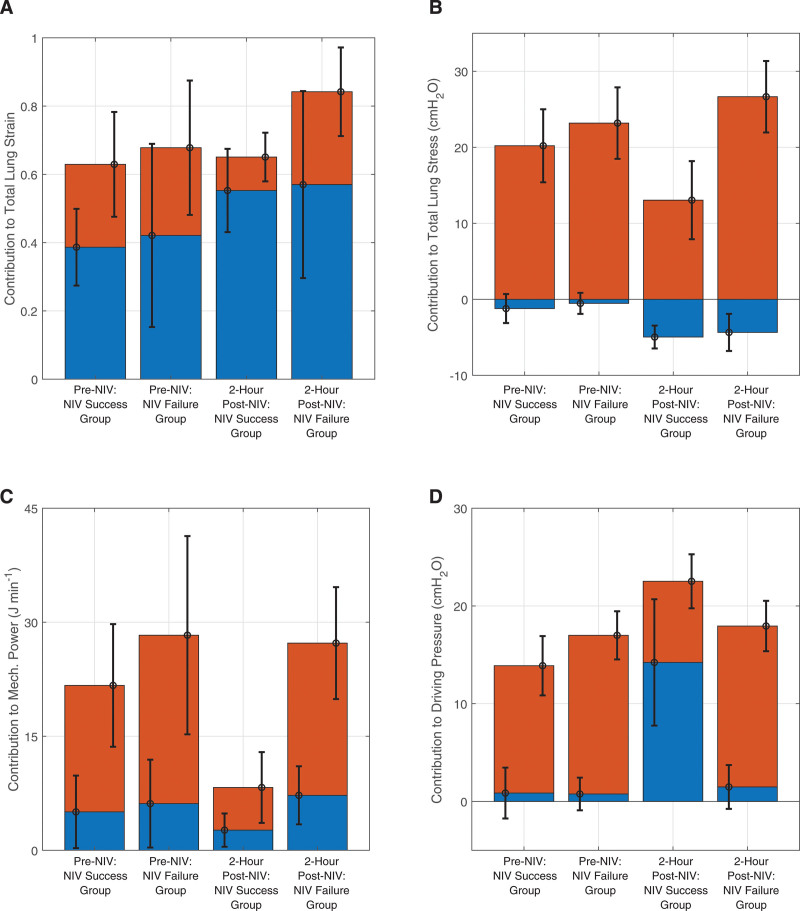
Mean contributions to lung injury indices from spontaneous respiratory effort (*red*) and external respiratory support (*blue*), with sds for each shown (*whisker*); contributions to the total lung strain (**A**), contributions to the total lung stress (**B**), contributions to the mechanical power (**C**), and contributions to the driving pressure (**D**). The sum of the *bars* is equal to the total in each case. NIV = noninvasive ventilation.

The relative contributions of the patients’ spontaneous respiratory efforts and the external respiratory support to total lung strain, total lung stress, driving pressure, and mechanical power are shown in **Figure 5**; and **Tables S15** and **S16** (http://links.lww.com/CCM/H553). In the digital twins of patients who were successful on NIV, large reductions were observed in the proportional contribution of spontaneous respiratory effort to total lung stress, total lung strain, mechanical power, and driving pressure. In contrast, no such reductions were observed in the digital twins of patients who failed NIV.

### Support Delivered by NIV in Success and Failure Groups

**Figure 6.**
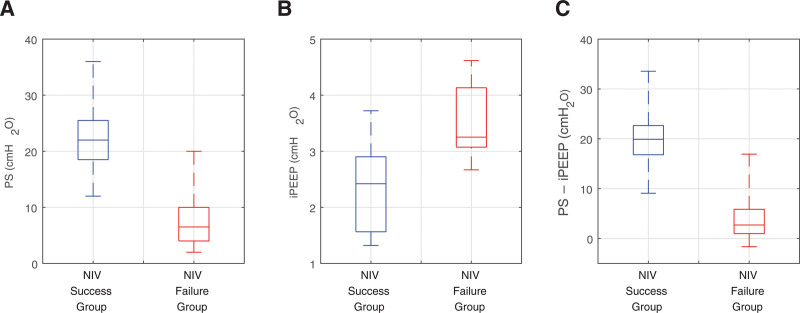
Comparison of the success and failure groups 2-hours after noninvasive ventilation (NIV) initiation; NIV inspiratory positive pressure support (PS) (**A**), the calculated intrinsic positive end-expiratory pressure (iPEEP) (**B**), and the difference between the inspiratory PS provided and the iPEEP (**C**).

Mean PEEP levels were similar during NIV (8.2 cm H_2_O, measured in patients) and HFNC (10.8 cm H_2_O on 60 L/min, calculated in the digital twins), suggesting that the primary additional support provided by NIV was the inspiratory positive PS above PEEP (mean 10 cm H_2_O, measured in patients). Mean PEEP settings were also similar between the NIV success and failure groups during the trial (**Fig. S4**, http://links.lww.com/CCM/H553). In contrast, mean PS was 63% lower in the failure group compared with the success group (**Fig. 6*A***), whereas intrinsic PEEP (iPEEP), which could be measured in the digital twins, was 52% higher in the failure group, compared with the success group (**Fig. 6*B***). As shown in **Figure 6*C***, this led to low levels of external PS (PS–iPEEP) being delivered to many patients in the failure group. The level of PS that could be applied in the failure group was constrained by the targets set for maximum values of Vt and RR—as shown in Figure S4, *B* and *C* (http://links.lww.com/CCM/H553), these targets are already being exceeded at the level of support provided, due to the patients’ continued high respiratory efforts.

## DISCUSSION

In this study, the creation of digital twins of a cohort of AHRF patients suggests that: 1) the additional respiratory support provided to these patients by NIV as compared with HFNC was primarily due to additional positive inspiratory pressure, 2) in patients who succeeded on NIV, this positive inspiratory pressure combined with PEEP produced alveolar recruitment and improved respiratory system compliance, leading to reduced respiratory effort, 3) the primary contribution to reductions in lung stress, strain, driving pressure, and mechanical power was due to lower patient respiratory effort, and 4) NIV failed in patients for whom only low levels of positive inspiratory PS could be applied without increasing the risk of patient self-inflicted lung injury (P-SILI) due to excessive Vts.

The significant correlation between ΔPes changes within the first 2 hours of NIV and radiographic progression at 24 hours reported in (6) suggested that P-SILI might be a potential mechanism of lung damage, which may contribute to outcome in these patients. However, the inability to measure standard indices of lung injury in these spontaneously breathing patients meant that this hypothesis could not be confirmed based on the original study data. We applied a novel methodology, based on the use of digital twins of the individual patients recruited to the study, in an attempt to “fill in the gaps” in the in vivo data.

Analysis of the data from the digital twins suggests that, before initiation of the NIV trial and when patients were receiving HFNC therapy, values of multiple indices of lung injury were at levels that would cause concern to clinicians if they could have been measured at the bedside (Fig. 2), indicating that this patient population might be at risk for failing noninvasive respiratory support due to P-SILI. This suggests that the generalized use of HFNC as a first line therapy in patients with moderate-to-severe AHRF requires continuous bedside assessment of respiratory mechanics and/or respiratory effort (17). PEEP levels provided by HFNC have not, to date, been measured at the bedside in AHRF patients, but could easily be calculated in the digital twins, where they were found to be similar (mean 10.8 cm H_2_O on 60 L/min) to levels measured in patients during the NIV trial (mean 8.2 cm H_2_O). The values of PEEP delivered by HFNC in the digital twins are higher than previous experimental studies that measured airway pressures produced in healthy volunteers (18). One possible explanation for this is that HFNC may produce higher PEEP in the “baby lungs” of AHRF/acute respiratory distress syndrome (ARDS) patients than in healthy volunteers, although further studies are required to confirm this hypothesis. High levels of PEEP, if accompanied by continued high spontaneous respiratory effort, could have the potential to cause lung injury, highlighting the need for close monitoring of patients receiving this support.

The additional inspiratory positive airway pressure provided by NIV as compared with HFNC therapy led to improved respiratory system compliance with minimal tidal recruitment (**Fig. S5**, http://links.lww.com/CCM/H553) in the digital twins of the patient subgroup for which NIV was successful. This could plausibly have allowed those patients to reduce their respiratory efforts, leading to large drops in the values of multiple indices of lung injury. In contrast, in the NIV failure group, our model suggests that only low levels of positive inspiratory PS could be applied without violating the limits on Vt and RR specified in the protocols for titrating the NIV settings. This level of support produced no change in compliance in the digital twins, which might have resulted in continued high respiratory efforts that, over time, could potentially lead to P-SILI.

It has been suggested previously that, in the presence of preexisting lung injury, transpulmonary pressure swings generated by elevated spontaneous respiratory efforts could be more harmful than the same swings generated via controlled mechanical ventilation (19). In the digital twins of patients receiving NIV who successfully avoided invasive mechanical ventilation, the relative contribution of spontaneous respiratory efforts to the total values of all lung injury indices was reduced.

It is of interest to note that results of this study align well with some of the proposed (but still debated) risk thresholds for the various indices of lung injury considered (12–15), with patients who went on to succeed on NIV generally having lung injury indices below the proposed threshold and those who failed having indices above. In the case of mechanical power and ΔP_pl_, the effect of NIV in the digital twins of the group for which it was successful was to reduce these indices from values well above their thresholds to values at the thresholds, while total lung stress was reduced from being at its threshold to being well below it (Fig. 2). In contrast, these indices of lung injury in the digital twins of the NIV failure group remained well above the proposed safety thresholds. Total lung strain was generally below the proposed risk thresholds, but increased more in the failure group. All the proposed risk thresholds have been developed using data from intubated and mechanically ventilated animal models or patients; to our knowledge, this is the first time they have been evaluated in the context of spontaneously breathing patients receiving noninvasive respiratory support.

Our results point to a potential role for iPEEP in contributing to the success or failure of NIV in some patients with AHRF. Although more usually associated with obstructive lung diseases, values of iPEEP of over 5 cm H_2_O have been measured in mechanically ventilated patients with ARDS (20), and high respiratory effort (as observed in many of the patients in [6]) is well known to have the potential to cause iPEEP (21). In our study, digital twins of the patients who failed NIV had higher levels of iPEEP. This, combined with the fact that these patients received generally lower levels of PS during the trial, suggests that many of these patients could have been receiving levels of external support that were inadequate to allow them to reduce their respiratory efforts, ultimately contributing to NIV failure.

This study has limitations. The digital twins were developed based on a relatively small number of patients from a single-center study (6). Due to the difficulty in measuring many key parameters in spontaneously breathing patients, there is likely to be some “noise” in the data on which the digital twins are based. Although the digital twins show excellent fidelity/matching to the actual patient data, they remain surrogates whose outputs are estimates of the values that would be produced in real patients. However, the mechanistic computational models on which the digital twins are based have been validated in numerous previous studies ([7–11] and references therein).

## CONCLUSIONS

Analysis of additional data provided by digital twins suggests that, in a cohort of patients with moderate-to-severe AHRF who had failed HFNC therapy, early inspiratory effort relief after initiation of NIV predicts success because it reflects improved lung mechanics caused primarily by additional positive inspiratory pressure. In the digital twins, the resulting reductions in respiratory effort lowered multiple indices associated with lung injury. In digital twins of patients who failed NIV, the levels of positive inspiratory PS that could be applied without producing excessive Vts were insufficient to enable reduction in patient respiratory effort. Although challenging to achieve in clinical practice, continuous detailed monitoring of patients’ respiratory efforts (perhaps with the aid of new technologies) may be the key to achieving success, and avoiding failure, in NIV.

## Supplementary Material
