# Supplementary material for: Digital Twins of Acute Hypoxemic Respiratory Failure Patients Suggest a Mechanistic Basis for Success and Failure of Noninvasive Ventilation
Source: Crit Care Med. 2024 May 29;52(9):e473–84. doi: 10.1097/CCM.0000000000006337 (PMC11321607; doi:10.1097/CCM.0000000000006337)
Supplement: Supplementary file 1 [file ccm-52-e473-s001.pdf]

Digital twins of acute hypoxemic respiratory failure patients suggest a mechanistic basis for success and failure of non-invasive ventilation

Critical Care Medicine

Liam Weaver, Hossein Shamohammadi, Sina Saffaran, Roberto Tonelli, Marianna Laviola, John G. Laffey, Luigi Camporota, Timothy E. Scott, Jonathan G. Hardman, Enrico Clini*, and Declan G. Bates

***Corresponding author:** enrico.clini@unimore.it (+39 059 4222918)

Supplementary Material

Table of Contents

Section 1 – ICSM Pulmonary Model 1

Section 2 – Modelling Spontaneous Breathing 10

Section 3 – Modelling Non-Invasive Respiratory Support 11

3.1 – Modelling High Flow Nasal Cannula Therapy 11

3.2 – Modelling Non-Invasive Ventilation 11

Section 4 – Calculating Lung Injury Indices 13

4.1 – Compliance 13

4.2 – Transpulmonary Pressure 13

4.3 – Pleural Pressure 14

4.4 – Baseline End Expiratory Lung Volume 14

4.5 – Total Lung Strain 14

4.6 – Total Lung Stress 14

4.7 – Mechanical Power 14

4.8 – Driving Pressure 15

4.9 – Lung Injury Thresholds 17

Section 5 – Patient Matching Protocol 18

5.1 – Matching Overview 18

5.2 – Input Data 18

5.3 – Cost Function 18

Section 6 – Patient Matching Tables 20

6.1 – Patient Characteristics..20

6.2 – HFNC Matching Results to Patient Data..21

6.3 – NIV Matching Results to Patient Data...22

Section 7 – Digital Twin Output Tables..23

7.1 – HFNC Results..23

7.1.1 – HFNC Respiratory Effort..23

7.1.2 – HFNC Blood-Gases...24

7.1.3 – HFNC Respiratory Mechanics...25

7.1.4 – HFNC Lung Injury Indices..26

7.2 – NIV Results...27

7.2.1 – NIV Respiratory Effort...27

7.2.2 – NIV Blood Gases...28

7.2.3 – NIV Respiratory Mechanics...29

7.2.4 – NIV Lung Injury Indices...30

Section 8 – Spontaneous Ventilation Contribution Isolation...31

8.1 – HFNC...31

8.2 – NIV..32

Section 9 – NIV Support Setting..33

Section 10 – Tidal Recruitment..34

References...35

Section 1 – ICSM Pulmonary Model

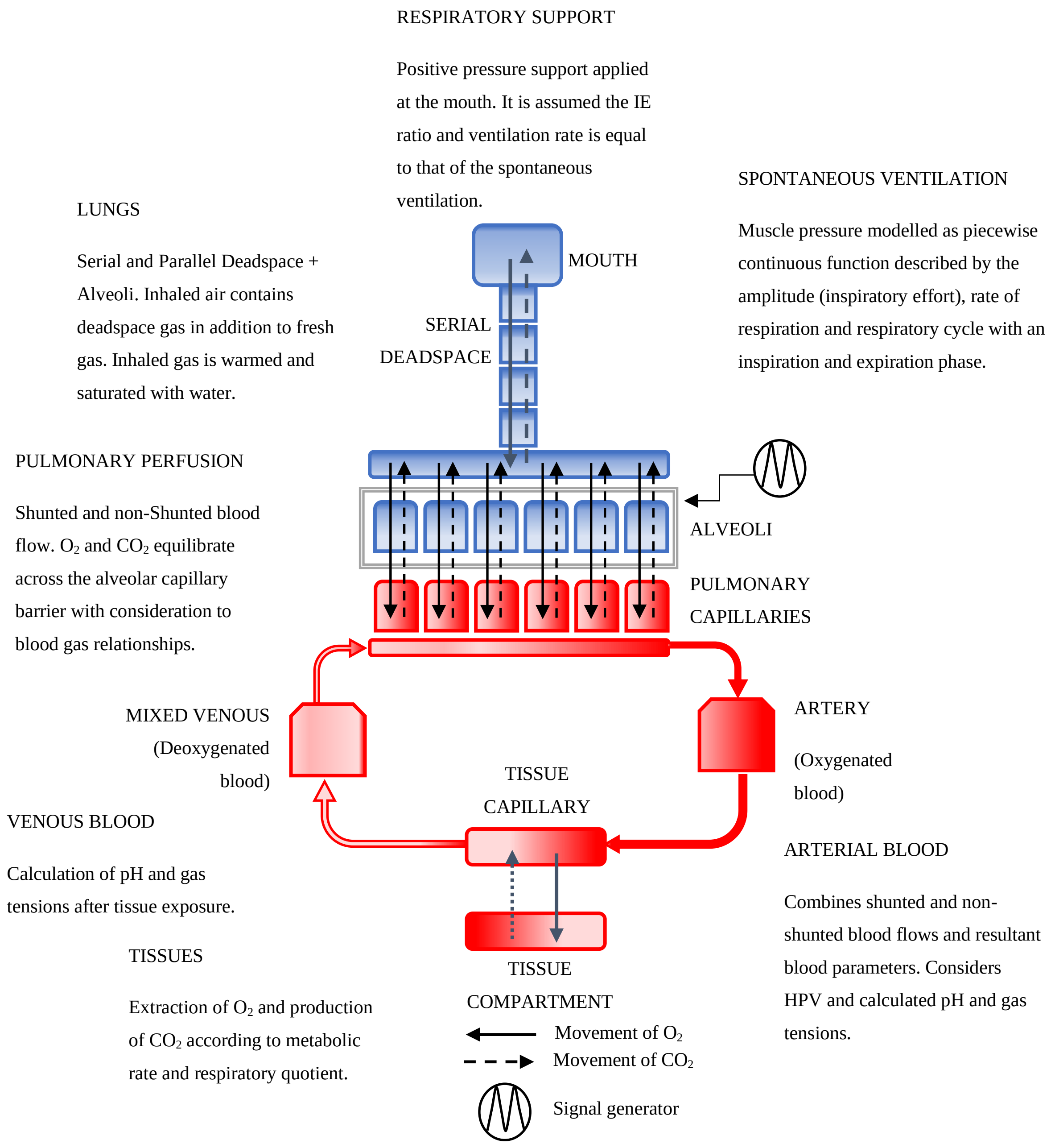

Figure S1 – Diagrammatic representation of the pulmonary model.

The model employed in this paper has been developed over the past several years and has been applied and validated on a number of different studies [E1]–[E10]. The model is organised as a system of several components (see Fig S1.1), each component representing different aspects of pulmonary dynamics and blood gas transport, e.g. the transport of air in the mouth, the tidal flow in the airways, the gas exchange in the alveolar compartments and their corresponding capillary compartment, the flow of blood in the arteries, the veins, the cardiovascular system, and the gas exchange process in the peripheral tissue compartments. Each component is described as several mass conserving functions and solved as algebraic equations, obtained or approximated from the published literature, experimental data and clinical observations. These equations are solved in series in an iterative manner, so that solving one equation at current time instant ($\mathrm{t_k}$) determines the values of the independent variables in the next equation. At the end of the iteration, the results of the solution of the final equations determine the independent variables of the first equation for the next iteration.

The iterative process continues for a predetermined time, *T*, representing the total simulation time, with each iteration representing a 'time slice' t of real physiological time (set to 10 ms). At the first iteration($\mathrm{t_k}, \mathrm{k} = 0$), an initial set of independent variables are chosen based on values selected by the user. The user can alter these initial variables to investigate the response of the model or to simulate different pathophysiological conditions. Subsequent iterations ($\mathrm{t_k} = \mathrm{t_{k-1}} + t$) update the model parameters based on the equations below.

The pulmonary model consists of the mechanical ventilation equipment, anatomical and alveolar dead space, anatomical and alveolar shunts, ventilated alveolar compartments and corresponding perfused capillary compartments. The pressure differential created by the mechanical ventilator or inspiratory muscles (i.e. when modelling spontaneous breathing) drives the flow of gas through the system. The series dead space (SD) is located between the mouth and the alveolar compartments and consists of the trachea, bronchi and the bronchioles where no gas exchange occurs. Inhaled gases pass through the SD during inspiration and alveolar gases pass through the SD during expiration. In the model, an SD of volume 60ml is split into 50 stacked layers of equal volumes (N_{SD} = 50). No mixing between the compartments of the SD is assumed.

Any residual alveolar air in the SD at the end of expiration is re-inhaled as inspiration is initiated. This residual air is composed of gases exhaled from both perfused alveolar compartments (normal perfusion) and the parallel dead space (PD) (alveolar compartments with limited perfusion). Therefore, the size of dead space (SD and PD) can have a significant effect on the gas composition of the alveolar compartments.

The inhaled air is initially assumed to consist of five gases: oxygen (O_2), nitrogen (N_2), carbon dioxide (CO_2), water vapor (H_2O) and a 5th gas (α) used to model additives such as helium or other anaesthetic

gases. During an iteration of the model, the flow (*f*) of air to or from an alveolar compartment *i* at time $t_k$is determined by the following equation:

$$f_i(t_k) = \frac{(p_v(t_k) - p_i(t_k))}{(R_u + R_{A,i})} \qquad \text{for } i = 1, \ldots, N_A \tag{1}$$

where $p_v(t_k)$ is the pressure at the mouth at (t_k), $p_i(t_k)$ is the pressure in the alveolar compartment i at (t_k), R_u is the constant upper airway resistance and $R_{A,i}$ is the bronchial inlet resistances of the alveolar compartment i. N_A is the total number of alveolar compartments (for the results in this paper, N_A = 100). The total flow of air entering the SD at time t_k is calculated by

$$f_{SD}(t_k) = \sum_{i=1}^{N_A} f_i(t_k) \tag{2}$$

During the inhaling phase, $f_{SD} \geq 0$, while in the exhaling phase $f_{SD} < 0$. During gas movement in the SD, the fractions of gases in the layer l of the SD, $F_{l,}$ $(l = 1, \ldots, N_{SD})$ is updated based on the composition of the total flow, f_{SD}, and the current composition of $F_{l,}$. If $f_{SD} \geq 0$, then air starts filling from the top layer (l = 1) to the bottom layer ($l = N_{SD}$); and vice versa for $f_{SD} < 0$.

The volume of gas x, in the ith alveolar compartment ($v_{i,x}$), is given by:

$$v_{i,x}(t_k) = \begin{cases} v_{i,x}(t_{k-1}) - f_i(t_k) \cdot \frac{v_{i,x}(t_{k-1})}{v_i(t_k)} & \textit{Exhaling} \\ v_{i,x}(t_{k-1}) + f_i(t_k) \cdot F_{N_{SD}}(t_k) & \textit{Inhaling} \end{cases} \qquad \text{for } i = 1, \ldots, N_A \tag{3}$$

In (3), x is any of the five gases (O_2, N_2, CO_2, H_2O or α). The total volume of the ith alveolar compartment, v_i is the sum of the volume of the five gases in the compartment.

$$v_i(t_k) = v_{i,O2}(t_k) + v_{i,N2}(t_k) + v_{i,CO2}(t_k) + v_{i,H2O}(t_k) + v_{i,\alpha}(t_k) \tag{4}$$

For the alveolar compartments, the tension at the centre of the alveolus and at the alveolar capillary border is assumed to be equal. The respiratory system has an intrinsic response to low oxygen levels in blood which is to restrict the blood flow in the pulmonary blood vessels, known as hypoxic pulmonary vasoconstriction (HPV). This is modelled as a mathematical function, resembling the stimulus response curve suggested by Marshall [11], and is incorporated into the simulator to gradually constrict the blood vessels as a response to low alveolar oxygen tension. The atmospheric pressure is fixed at 101.3 kPa and the body temperature is fixed at 37.2°C.

At each t_k, equilibration between an alveolar compartment and the corresponding capillary compartment is achieved iteratively by moving small volumes of each gas between the compartments until the partial pressures of these gases differ by <1% across the alveolar-capillary boundary. The process includes the nonlinear movement of O_2 and CO_2 across the alveolar capillary membrane during equilibration.

In blood, the total O_2 content (C_{O2}) is carried in two forms, as a solution and as oxyhaemoglobin (saturated haemoglobin):

$$C_{O2}(t_k) = S_{O2}(t_{k-1}) \cdot Huf \cdot Hb + P_{O2}(t_{k-1}) \cdot O_{2sol} \quad (5)$$

In this equation, S_{O2} is the haemoglobin saturation, *Huf* is the Hufner constant, Hb is the haemoglobin content and O_{2sol} is the O_2 solubility constant. The following pressure-saturation relation, as suggested by [12] to describe the O_2 dissociation curve, is used in this model:

$$S_{O2}(t_k) = \left(\left(\left(P_{O2}^3(t_{k-1}) + 150 \cdot P_{O2}(t_{k-1})\right)^{-1} \times 23400\right) + 1\right)^{-1} \quad (6)$$

S_{O2} is the saturation of the haemoglobin in blood and $P_{O2}$is the partial pressure of oxygen in the blood. As suggested by [13], P_{O2} has been determined with appropriate correction factors in base excess BE, temperature T and pH (7.5005168 = pressure conversion factor from kPa to mmHg):

$$P_{O2}(t_k) = 7.5006168 \cdot P_{O2}(t_{k-1}) \cdot 10^{[0.48(pH(t_{k-1})-7.4)-0.024(T-37)-0.0013 \cdot BE]} \quad (7)$$

The CO_2 content of the blood (C_{CO2}) is deduced from the plasma CO_2 content ($C_{CO2plasma}$) [14] by the following equation:

$$C_{CO2}(t_k) = C_{CO2plasma}(t_{k-1}) \cdot \left[1 - \frac{0.0289 \cdot Hb}{(3.352-0.456 . S_{O2}(t_k)) \cdot (8.142-pH(t_{k-1}))}\right] \quad (8)$$

where S_{O2} is the O_2 saturation, Hb is the haemoglobin concentration and pH is the blood pH level. The coefficients were determined as a standardized solution to the McHardy version of Visser's equation [15], by iteratively finding the best fit values to a given set of clinical data. The value of $C_{CO2plasma}$ is deduced using the Henderson-Hasselbach logarithmic equation for plasma C_{CO2} [16]:

$$C_{CO2plasma}(t_k) = 2.226 \cdot s_{CO2} \cdot P_{CO2}(t_{k-1}) \left(1 + 10^{(pH(t_{k-1}) - pK')}\right) \quad (9)$$

where s_{CO2} is the plasma CO_2 solubility coefficient and pK' is the apparent pK (acid dissociation constant of the CO_2 bicarbonate relationship). P_{CO2} is the partial pressure of CO_2 in plasma and '2.226' refers to the conversion factor from millimoles per liter to ml/100ml. [16] gives the equations for s_{CO2} and pK' as:

$$s_{CO2} = 0.0307 + 0.0057 \cdot (37 - T) + 0.00002 \cdot (37 - T)^2 \quad (10)$$

$$pK' = 6.086 + 0.042 \cdot (7.4 - pH(t_{k-1})) + (38 - T) \cdot \left(0.00472 + \left(0.00139 - (7.4 - pH(t_{k-1}))\right)\right) \quad (11)$$

$P_{CO2}(t_k)$ is determined by incorporating the standard Henry's law and the s_{CO2} (the CO_2 solubility coefficient above). For pH calculation, the Henderson Hasselbach and the Van Slyke equation [17] are

combined. Below is the derivation of the relevant equation. The Henderson-Hasselbach equation (governed by the mass action equation (acid dissociation)) states that:

$$\text{pH} = \text{pK} + \log\left(\frac{bicarbonateconcentration}{carbonicacidconcentration}\right) \tag{12}$$

Substituting pK=6.1 (under normal conditions) and the denominator $(0.225 \cdot P_{CO2})$ (acid concentration being a function of CO_2 solubility constant 0.225 and P_{CO2} (in kPa)) gives:

$$\text{pH}(t_k) = 6.1 + \log\left(\frac{HCO_3(t_{k-1})}{0.225 \cdot P_{CO2(t_k)}}\right) \tag{13}$$

For a given pH, base excess (BE), and haemoglobin content (Hb), HCO_3 is calculated using the Van-Slyke equation, as given by [17]:

$$HCO_3(t_k) = \left((2.3 \times \text{Hb} + 7.7) \times (\text{pH}(t_k) - 7.4)\right) + \frac{\text{BE}}{(1-0.023 \times \text{Hb})} + 24.4 \tag{14}$$

The capillary blood is mixed with arterial blood using the equation below which considers the anatomical shunt (Sh) with the venous blood content of gas x ($C_{v,\,x}$), the non-shunted blood content from the pulmonary capillaries ($C_{cap,\,x}$), arterial blood content ($C_{a,\,x}$), the arterial volume (v_a) and the cardiac output (CO).

$$C_{a,\,x}(t_k) = \frac{CO(t_k) \cdot \left(Sh \cdot C_{v,\,x}(t_k) + (1-Sh) \cdot C_{cap,\,x}(t_k)\right) + C_{a,\,x}(t_k) \cdot (v_a(t_k) - CO(t_k))}{v_a(t_k)} \tag{15}$$

The peripheral tissue model consists of a single tissue compartment, acting between the peripheral capillary and the active tissue (undergoing respiration to produce energy). The consumed O_2 (V_{O2}) is removed and the produced CO_2 (V_{CO2}) is added to this tissue compartment. As per the alveolar equilibration, peripheral capillary gas partial pressures reach equilibrium with the tissue compartment partial pressures, with respect to the nonlinear movement of O_2 and CO_2. Metabolic production of acids, other than carbonic acid via CO_2 production, is not modelled. After peripheral tissue equilibration of gases, the venous calculations of partial pressures, concentrations and pH calculations are done using comparable equations to those above.

A simple equation of renal compensation for acid base disturbance is incorporated. The base excess (BE) of blood under normal conditions is zero. BE increases by 0.1 per time slice if pH falls below 7.36 (to compensate for acidosis) and decreases by 0.1 per time slice if pH rises above 7.4 (alkalosis).

Each alveolar compartment has a unique and configurable alveolar compliance, alveolar inlet resistance, vascular resistance, extrinsic (interstitial) pressure, threshold opening pressure and threshold closing pressure. For the ith compartment of N alveolar compartments, the pressure p_i is determined by:

$$p_i(t_k) = S_i(v_i(t_k) - V_c)^2 + P_{ext,i} v_i(t_k) + P_{INSP} \quad \text{for } i = 1, \ldots, N_A \tag{16}$$

where

$$S_i = k_i {N_A}^2/200000 \quad \text{and} \quad V_c = 0.2V_{FRC}/N_A$$

Equation (16) determines the alveolar pressure p_i (as the pressure above atmospheric in cmH_2O) for the ith compartment of N_A number of alveolar compartments for the given volume of alveolar compartment, $v_i(t)$ in millilitres. The alveolar compartments are arranged in parallel and interact with the series dead space with respect to the movement of gases. The use of the square of the difference between v_i and Vc causes alveolar pressure to increase at volumes below Vc, leading to exhalation and a tendency to "snap shut" (mathematical note: the pressure with respect to volume is thus a U-shaped curve) [18].

P_{ext} (per alveolar unit, in cmH_2O) represents the *effective net pressure* generated by the sum of the effects of factors outside each alveolus that act to distend that alveolus; positive components include the outward pull of the chest wall, and negative effects include the compressive effect of interstitial fluid in the alveolar wall. Incorporating P_{ext} in the model allows us to replicate the situation of alveolar units that have less structural support or that have interstitial oedema, and thus have a greater tendency to collapse. A negative value of P_{ext} indicates a scenario where there is compression from outside the alveolus causing collapse. The parameter S_i is a scalar that determines the intra-alveolar pressure for a given volume (with respect to a constant collapsing volume V_c) and is dependent on the parameter k. The units of S_i are $cmH_2O\ ml^{-2}$. P_{INSP}, which represents the pressure generated by the respiratory muscles acting on the lung, is described in the next section. Finally, V_c is defined as a "constant collapsing volume" at which the alveolus tends to empty (through Laplace effects) and represents a fundamental mechanical property of tissue and surfactant [18]. V_{FRC} is the end expiratory volume of the lungs.

The effect of the three parameters on the volume–pressure relationship of the alveolar compartments can be observed in Figure S2.

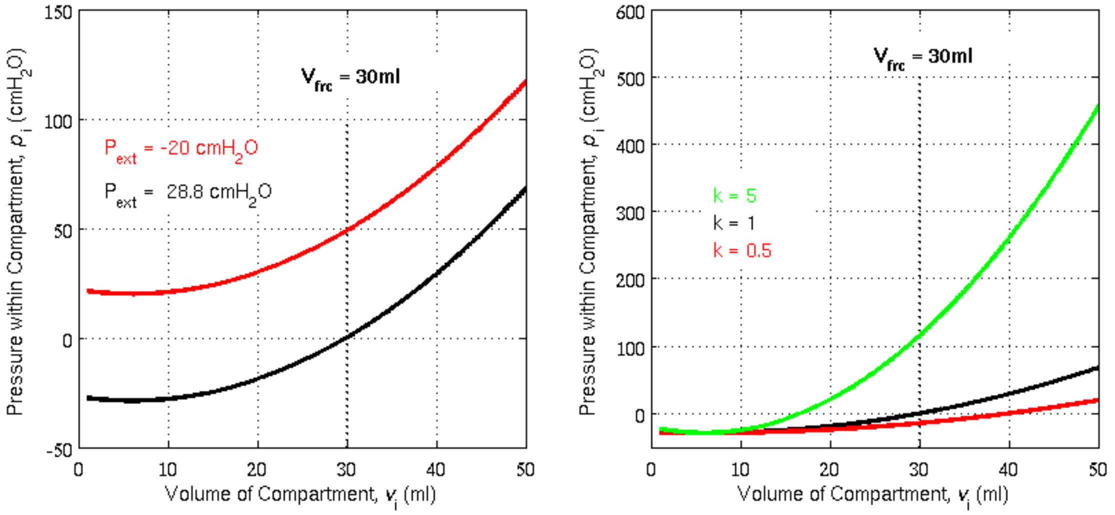

Figure S2 – The effect of varying the parameters of Equation (18) on the pressure volume relationship of the model (P_{INSP} = 0) and under mechanical ventilation.

The nominal values for ($P_{ext,i}$, S_i) have been determined such that at the end of expiration, the alveolar pressure within the compartment is also equal to zero, i.e. at an pre-set value of functional residual capacity.

We consider each of the parameters mentioned above ($P_{ext,i}, S_i$) to be different yet essential components for representing a diseased lung that affect the volume pressure relationship of the alveolar compartments. For example, for a given volume v_i, increasing S_i increases the corresponding alveolar pressure of the alveolar compartment. When compared to another compartment with a lower S_i, a larger pressure gradient would be needed to drive air into the compartment; thus effectively the compartment will be behaving as a stiffer lung unit.

Decreasing $P_{ext,i}$ decreases the alveolar pressure such that the pressure gradient (especially during exhaling) forces the air out of the alveolar compartment until the volume of the compartment collapses (v_i = 0 ml). Note that, in effect, the parameters are influencing the resting volume of the compartments (when the alveolar pressure, p_i, is equal to zero).

In the model, the airway resistance R_{aw} is determined by the following equation for N parallel compartments:

$$R_{aw} = R_U + R_{L_{aw}} \tag{17}$$

In the above equation, the lower airway resistance, $R_{L_{aw}}$, is determined by the following:

$$\frac{1}{R_{aw}} = \frac{1}{R_{A,1}} + \frac{1}{R_{A,2}} + \cdots + \frac{1}{R_{A,N_A}}, \text{ for } i = 1, \dots, N_A$$

where $R_{A,i}$ is the bronchial inlet resistance of the ith compartment, which is defined by:

$$R_{A,i} = m_i R_{A0}$$

where R_{A0}corresponds to the default bronchial inlet resistance of an alveolar compartment. R_{A0} is set to $10^{-7} \times N_A$ for a healthy lung (the inlet resistance is higher for a model with more compartments as the volume of each compartment decreases). m_i is a coefficient of the airway resistance, representing a dynamic change in airway resistance and is determined by the equation:

$$m_i = \begin{cases} 1, & \mathrm{t}_{\mathrm{o},i} \le 0 \\ 10^{10}, & \mathrm{t}_{\mathrm{o},i} > 0 \end{cases} \quad \text{for} i = 1, \dots, N_A \tag{18}$$

where,

$$\mathrm{t}_{\mathrm{o},i} = \begin{cases} \mathrm{t}_{\mathrm{o},i} - t\,, & p_{trachea} \ge \mathrm{TOP}_i \\ \tau_{c,i}, & p_{trachea} < \mathrm{TOP}_i \end{cases} \quad \text{for} i = 1, \dots, N_A \tag{19}$$

$p_{trachea}$ is the pressure in the trachea and TOP_i is a value between 5 and 50 cmH_2O for the ith alveolar compartment. Additionally, a threshold opening pressure (TOP) at low lung volumes needs to be attained for a collapsed alveolar unit to open. Recruitment is a time dependent process, with different airways recruiting at different times, once the threshold pressure has been achieved [19], [20]. The equations within the model are solved iteratively as a discretised system. Each iteration represents a physiological time slice of t (10 ms). The time-dependent recruitment phenomenon is achieved in the model by the introduction of a parameter t_o. For collapsed compartments, t_o is set to τ_c which represents the time it could take for collapsed alveoli to open after a threshold pressure is reached. Once $p_{trachea} \ge \mathrm{TOP_i}$ is satisfied, the counter t_o decrements during every iteration, and triggers the opening of the airway (m_i= 1) as $\mathrm{t}_\mathrm{o} \le 0$. Otherwise m_i is set to a high value (10^{10}) to represent a collapsed airway.

N_A (the number of alveolar compartments) is fixed and set by the user (i.e. they do not change during a simulation). Therefore, during a simulation, m_i, chiefly represents the relatively small changes in inlet resistance during tidal ventilation. Furthermore, R_{B0} are also preset and fixed, and do not change during the simulation. The only change in airway resistance which is dynamic is m_i which is dependent on the volume v_i at time($\mathrm{t_k}$).

Finally, the pulmonary vascular resistance PVR is determined by

$$\frac{1}{PVR} = \frac{1}{R_{V,1}} + \frac{1}{R_{V,2}} + \cdots + \frac{1}{R_{V,N_A}}, \text{ for } i = 1, \dots, N_A \tag{20}$$

where the resistance for each compartment $R_{V,i}$ is defined as

$$R_{V,i} = \delta_{Vi} R_{V0} \tag{21}$$

R_{V0} is the default vascular resistance for the compartment with a value of $160 \cdot N_A$ dynes s cm^{-5} min^{-1}, and δ_{Vi} is the vascular resistance coefficient, used to implement the effect of hypoxic pulmonary vasoconstriction.

The net effect of these components of the simulation is that the defining, clinical features of acute respiratory disease may be observed in the model: alveolar gas-trapping (with intrinsic PEEP), collapse-reopening of alveoli (with gradual reabsorption of trapped gas if re-opening does not occur), limitation of expiratory flow etc.

Section 2 – Modelling Spontaneous Breathing

The pressure at the airway opening (i.e. mouth) is relatively constant at atmospheric pressure, while the alveolar pressure value varies during inspiration and expiration. The active contraction of the respiratory muscles during inspiration generates a negative alveolar pressure relative to the atmospheric pressure. The variable P_{INSP}, which represents the pressure generated by the respiratory muscles acting on the lung, is modelled as a piecewise function as described in [21] and adapted from [22]. The function consists of a parabolic profile during the inspiration phase of the respiratory cycle, representing the progressive increase in pressure exerted by the respiratory muscles, followed by an exponential profile during the expiration phase of the respiratory cycle, characterizing the passive relaxation of the muscles. During a single respiratory cycle, P_{INSP} at time $\mathrm{t_k}$, is calculated as

$$P_{INSP}(\mathrm{t_k}) = \begin{cases} \dfrac{-P_{MIN}}{T_I.T_E}.\mathrm{t_k}^2 + \dfrac{P_{MIN}.T}{T_I.T_E}.\mathrm{t_k} & \mathrm{t_k} \in [0, T_I] \\ \dfrac{P_{MIN}}{1 - e^{-\frac{T_E}{\tau}}}.\left(e^{-\frac{((\mathrm{t_k}-T_I)}{\tau}} - e^{-\frac{T_E}{\tau}}\right) & \mathrm{t_k} \in [T_I, T] \end{cases}$$

P_{INSP} decreases from zero to its minimum end-inspiratory value (P_{MIN}) (i.e., maximum effort) during inspiration and returns to zero at end of expiration. T is calculated from the set respiratory rate, RR, (T = 60/RR). T_I and T_E are the duration of inspiration and expiration, such that (T = $T_I + T_E$). T_I is calculated from ($T_I = T * DC$) where DC is the duty cycle, set to 0.33. τ is the time constant of the expiratory profile and is set to Te/RR.

The resulting alveolar pressure p_i depends on P_{INSP}, as well as on the pressures of the gases within the compartment, the stiffness of the compartment (S_i), and parameter P_{ext} which represents extrinsic pressures acting on the compartment.

Section 3 – Modelling Non-Invasive Respiratory Support

3.1 – Modelling High Flow Nasal Cannula Therapy

To accurately recreate the effects of high flow nasal cannula (HNFC) therapy, we need to consider the differing effects on pressure dynamics during both the inspiratory and expiratory phases. When HFNC is applied, the pressure at the nares increases, primarily attributed to the HFNC-induced augmented resistance during exhalation, resulting in PEEP.

To recreate this phenomenon in our model, we calculate the corresponding increase in pressure by multiplying the HFNC flow by the subject's total airway resistance (R_{aw}) (Eq. 22). The modelled R_{aw} comprises two main respiratory airway resistances which are located in series. The first resistance, the upper airways ($R_{U, aw}$), represents the nasal cavity, oral cavity, and trachea whilst the second resistance, the lower airways (R_L, aw) represents the bronchi and the bronchioles, and the inlet resistances for multiple alveoli which make up the lungs, placed in parallel.

To simulate the effect of HFNC during the inspiratory phase, we apply an effective pressure at the end of the upper airways (i.e., at the end of the trachea) by calculating the pressure drop which occurs between the mouth and lungs (Eq. 23). During the expiratory phase, this flow is reversed, with lung pressure driving the flow and mouth pressure opposing it (Eq. 24).

$$P_{mouth} = P_{atm} + (flow\ rate \times R_{aw}) \tag{22}$$

$$P_{trachea} = P_{mouth} - \left(\frac{P_{mouth} - P_{lungs}}{R_{aw}} \times R_{U,aw}\right) \tag{23}$$

$$P_{trachea} = P_{lungs} - \left(\frac{P_{lungs} - P_{mouth}}{R_{aw}} \times R_{L,aw}\right) \tag{24}$$

3.2 – Modelling Non-Invasive Ventilation

During Non-Invasive Ventilation (NIV), two pressures are provided to the patient in addition to their spontaneous respiratory efforts: positive end expiratory pressure (PEEP) during exhalation, and an additional inspiratory pressure support (PS) is provided above PEEP during inhalation. To accurately simulate the effect of PEEP and PS, the tracheal pressure applied factors in the pressure drop which occurs due to resistances within the airways.

To replicate the effect of breath triggering in the support being received by the patient, PS is only provided to the patient once their lung pressure falls below the set PEEP due to the negative pressure generated by the respiratory muscles. This pressure then lasts throughout the rest of the active inspiratory phase, with the supporting pressure reducing to PEEP at the beginning of the patient expiratory phase. There is assumed to be no asynchrony between the patient's spontaneous respiratory efforts and the support they are receiving.

Section 4 – Calculating Lung Injury Indices

The following subsections list the methodology for the calculation of each of the key indices of lung injury presented in this study.

4.1 – Compliance

The respiratory system compliance (C_{rs}) is calculated using:

$$C_{rs} = \frac{VT}{\Delta P_{lungs}}$$

where VT is the tidal volume, and ΔP_{lungs} is the pressure swing between peak lung pressure and minimum lung pressure. Note that this equation gives the respiratory system compliance, and not the lung compliance, as we are applying P_{INSP} and P_{ext} directly to the alveolar compartments.

To calculate the lung compliance (C_L) we use the elastance equation:

$$\frac{1}{C_L} = \frac{1}{C_{rs}} - \frac{1}{C_{cw}}$$

where C_{cw} is the chest wall compliance, estimated to be 4% of the vital capacity [23].

Vital capacity is estimated using the following equations [24]:

$$VC_{male} = 27.63 - (0.112 \times Age) \times Height$$

$$VC_{female} = 21.78 - (0.101 \times Age) \times Height$$

4.2 – Transpulmonary Pressure

The transpulmonary pressure (P_L) was calculated as:

$$P_L = \frac{\Delta V}{C_L}$$

where C_L is the dynamic lung compliance, and ΔV is the difference between the current lung volume and the unstressed lung volume (the residual volume, V_{RV}).

The residual volume was estimated for each patient using the following equations [25]:

$$V_{RV,male} = (1.31 \times Height) + (0.022 \times Age) - 1.23$$

$$V_{RV,female} = (1.81 \times Height) + (0.016 \times Age) - 2$$

4.3 – Pleural Pressure

The pleural pressure (P_{pl}) was derived from the transpulmonary and lung pressures (P_L):

$$P_{pl} = P_{lungs} - P_L$$

4.4 – Baseline End Expiratory Lung Volume

To calculate the total strain being applied to the lungs, we first need to determine a baseline end-expiratory lung volume against which we will compare the change in inspired lung volume. To estimate this, each of the digital twins had their respiratory rate set to 14 bpm and their muscle pressure adjusted to achieve a tidal volume set to 7 ml/kg of predicted body (to emulate quiet breathing). The end-expiratory lung volume ($EELV$) was then recorded and used as $EELV_{Baseline}$.

4.5 – Total Lung Strain

The total lung strain was calculated as the sum of the static and dynamic strains across the lung [26]. The dynamic and static strains are calculated as follows:

$$Dynamic\ Strain = \frac{VT}{EELV_{Baseline}}$$

$$Static\ Strain = \frac{EELV - EELV_{Baseline}}{EELV_{Baseline}}$$

$$Total\ Strain = Dynamic\ Strain + Static\ Strain$$

4.6 – Total Lung Stress

The total stress applied to the lungs is, by definition, the stress (pressure) applied to the lungs attributed to the difference between the unstressed volume of the lung, V_{RV}, and the current volume of the lung. Therefore, the total lung stress is equal to the transpulmonary pressure swing (ΔP_L) [27].

$$\Delta P_L = P_{L,max} - P_{L,min}$$

4.7 – Mechanical Power

The energy applied to the lungs per breath is equal to the area enclosed by the pressure-volume curve. To determine the energy applied per minute (the power), we multiply this area by the respiratory rate and apply a unit conversion factor, as described in [28]:

$$Power = \left(0.5 \times VT \times \Delta P_{tp}\right) \times RR \times 0.0000980665$$

4.8 – Driving Pressure

The concept of driving pressure (ΔP), whilst being well defined in invasive mechanical ventilation as the difference between plateau pressure (P_{plat}) and $PEEP$, is a more challenging concept in spontaneous ventilation due to the absence of a satisfactory P_{plat}. It has been proposed that ΔP can be approximated in spontaneously breathing patients receiving non-invasive respiratory support by using an inspiratory hold to get an estimate of P_{plat} [29], but this is still an approximation of the true ΔP which is generating the inspiratory flow.

Within the digital twins, we are able to calculate the change in lung pressure which occurs during the inspiratory phase as a result of the support and the patient's inspiratory efforts, allowing an accurate calculation of the pressure which is distending the lung.

In the absence of an elevated inspiratory PS (e.g., during HFNC, CPAP, or unassisted spontaneous ventilation), ΔP can be calculated as the difference between the end-expiratory lung pressure ($EELP$) and the minimum lung pressure ($P_{lung,min}$) (Figures S3a and S3b). In support modalities which deliver an inspiratory PS above the level of PEEP set, the elevated inspiratory mouth pressure must be factored in. To do so, we measure ΔP as the difference <u>between</u> the mouth pressure (P_{mouth}) and $P_{L,min}$ (Figure S3c). This is summarised below:

$$\Delta P = \begin{cases} EELP - P_{lung,min} & PS = 0 \\ \mathrm{P_{mouth}} - P_{lung,min} & PS \neq 0 \end{cases}$$

The consequence of the above is that when an inspiratory PS is present, the support increases the total distending pressure being applied to the lung. This then facilitates a patient reducing their spontaneous respiratory effort whilst maintaining ΔP.

While the above two cases are the most likely to occur and hold true in most cases, there is a third case which can occur. If the PS provided to a patient above PEEP fails to exceed the patients $EELP$ due to the presence of intrinsic PEEP ($iPEEP$), then the support will fail to augment the ΔP. In these cases, most likely to occur when the PS provided is very low, the distending pressure of the lung remains the difference between $EELP$ and $P_{L,min}$, and the patient is unable to reduce their spontaneous respiratory effort without seeing a reduction to ΔP (Figure S3d).

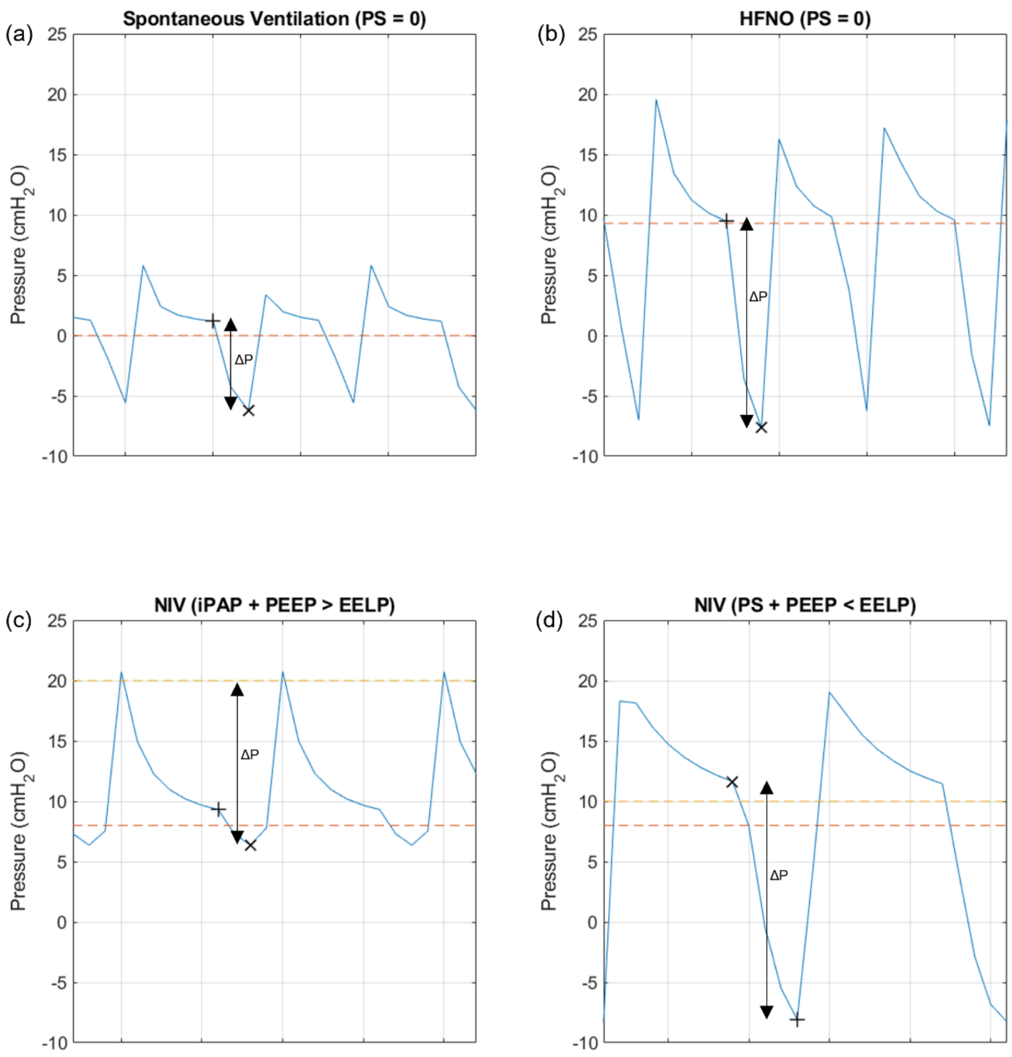

Figure S3 – Four driving pressure instances which can occur. The lung pressure is in blue, with the PEEP in orange (dashed) and the PS + PEEP in yellow (dashed). In each case, the end-expiratory lung pressure (EELP) is signified with a cross (x), and the minimum lung pressure ($P_{lung,min}$) with a plus (+). Each plot has an arrow showing the driving pressure measured. In cases (a) and (b), no PS+PEEP line is present as PS = 0 cmH_2O.

4.9 – Lung Injury Thresholds

	**Lower Risk**	**Higher Risk**
**Total Lung Strain**	< 1.29	> 1.5
**Mechanical Power** ($J\ min^{-1}$)	< 7	> 12
**Δ Pleural Pressure** (cmH_2O)	< 8	> 12
**Total Lung Stress** (cmH_2O)	< 17.4	> 20.25
**Driving Pressure** (cmH_2O)	< 12	> 15

Table S1 - Potential Lung Injury Safety Thresholds, based on data in [30], [31], [32], [33]

Section 5 – Patient Matching Protocol

5.1 – Matching Overview

Each “digital twin” is a computation model which is designed to reflect the measured characteristics of one of the patients from [34]. The twin is created by the careful calibration of the model parameters which govern the system functionality by the minimisation of a user defined cost function (see Section 5.3).

5.2 – Input Data

Clinical Data	Unit	Use
Age	Years	Estimation of residual volume and chest wall compliance
Height	Meters	Estimation of residual volume and chest wall compliance
Gender	-	Estimation of residual volume and chest wall compliance
Weight	Kilograms	Direct model input
Fraction of inspired oxygen	%	Direct model input
Respiratory rate	Breaths/minute	Direct model input
HFNC Flow Rate	Litres/minute	Direct model input (used during HFNC only)
NIV Pressure Support	cmH_2O	Direct model input (used during NIV only)
NIV PEEP	cmH_2O	Direct model input (used during NIV only)

Table S2 – Clinical data which is used to create the digital twins

5.3 – Cost Function

The cost function aims to minimise the error between the simulator output and the clinical observations by adapting the calibration of the model parameters. The data used in the cost function can be found in Table S3.

Data	Unit
PaO_2	mmHg
$PaCO_2$	mmHg
Tidal esophageal pressure swing	cmH_2O
Tidal Volume	ml

Table S3 – Data used within cost function

Section 6 – Patient Matching Tables

6.1 – Patient Characteristics

Digital Twin ID (DT ID)	Diagnosis (0= pneumonia, 1= ARDS, 2= sepsis/other)	Age (Years)	Sex (F = 0, M =1)	Weight (kg)	Predicted Body Weight (kg)	Height (m)	Chest Wall Compliance (ml/cmH2O)	Calculated Residual Volume (L)	NIV Outcome (Success = 1, Failure = 0)
1	1.00	81	1	52.00	44.18	1.46	108.38	2.46	1
2	1.00	81	1	94.50	76.03	1.81	134.36	2.92	0
3	2.00	71	0	68.00	69.71	1.79	104.60	2.38	1
4	0.00	72	0	114.00	79.72	1.90	110.26	2.59	0
5	2.00	81	1	64.00	43.27	1.45	107.64	2.45	1
6	1.00	68	0	77.00	52.42	1.60	95.44	1.98	1
7	1.00	49	1	84.50	77.85	1.83	162.08	2.25	0
8	0.00	93	1	57.00	64.20	1.68	115.68	3.02	0
9	1.00	81	1	61.00	65.11	1.69	125.45	2.77	1
10	1.00	68	1	113.00	82.40	1.88	150.51	2.73	1
11	0.00	67	1	104.00	79.67	1.85	148.93	2.67	1
12	1.00	62	0	83.00	78.81	1.89	117.32	2.41	0
13	1.00	79	1	89.50	87.86	1.94	145.75	3.05	0
14	0.00	71	1	86.00	74.21	1.79	140.89	2.68	1
15	0.00	63	1	95.00	65.11	1.69	139.08	2.37	0
16	1.00	84	0	84.00	72.44	1.82	96.79	2.64	1
17	1.00	81	1	80.00	57.83	1.61	119.51	2.66	1
18	0.00	73	0	114.00	73.35	1.83	105.46	2.48	1
19	0.00	52	0	96.00	62.43	1.71	113.05	1.93	1
20	1.00	68	1	76.50	69.66	1.74	139.30	2.55	0
21	0.00	71	1	98.00	76.94	1.82	143.26	2.72	1
22	1.00	82	1	91.50	84.22	1.90	140.19	3.06	1
23	0.00	83	0	59.50	56.97	1.65	88.42	2.31	0
24	1.00	64	1	65.00	75.12	1.80	147.33	2.54	0
25	0.00	73	1	88.70	79.67	1.85	143.96	2.80	1

Table S4

6.2 – HFNC Matching Results to Patient Data

Digital Twin ID (DT ID)	Resp. Rate (bpm)	FiO2 (%)	PaO2 (mm Hg)			pCO2 (mmHg)			dPes VS dPpl (cmH2O)			Tidal Volume (ml)		
			Data	Simulation	% Error	Data	Simulation	% Error	Data	Simulation	% Error	Data	Simulation	% Error
1	57.00	45.00	87.30	84.91	2.74	31.00	29.13	6.03	40.00	42.44	6.10	480.70	365.20	24.03
2	28.00	60.00	67.50	67.32	0.26	34.00	33.96	0.12	32.00	32.15	0.46	616.00	608.90	1.15
3	45.00	50.00	72.00	71.95	0.07	31.00	30.93	0.23	49.00	49.03	0.06	840.00	821.20	2.24
4	34.00	45.00	76.50	76.49	0.01	39.80	39.73	0.18	42.00	42.04	0.10	751.00	747.60	0.45
5	50.00	45.00	60.30	60.19	0.18	31.00	30.86	0.45	27.00	27.82	3.04	452.00	430.80	4.69
6	46.00	55.00	78.57	79.59	1.29	25.00	24.06	3.76	38.00	37.85	0.39	697.00	572.00	17.93
7	34.00	60.00	60.00	59.98	0.03	36.00	35.92	0.22	38.00	38.41	1.08	882.00	867.90	1.60
8	42.00	60.00	76.00	75.96	0.05	29.00	28.71	0.99	42.00	43.56	3.72	875.00	819.96	6.29
9	42.00	85.00	58.40	59.52	1.91	35.00	34.40	1.72	28.00	32.60	16.41	786.00	591.75	24.71
10	20.00	55.00	73.70	73.87	0.23	44.00	43.97	0.06	21.00	21.62	2.94	656.00	634.59	3.26
11	28.00	50.00	66.25	66.17	0.12	42.00	41.77	0.55	31.00	31.15	0.48	800.00	790.63	1.17
12	26.00	70.00	70.00	69.59	0.58	19.00	18.71	1.50	38.00	38.21	0.55	738.00	700.06	5.14
13	25.00	40.00	90.00	89.91	0.10	34.00	33.95	0.14	32.00	32.05	0.17	1068.00	1060.40	0.71
14	26.00	70.00	93.33	92.97	0.39	44.00	41.25	6.25	22.00	23.58	7.18	672.00	599.30	10.82
15	38.00	60.00	60.00	59.96	0.07	42.00	42.01	0.02	42.00	41.97	0.07	852.00	851.47	0.06
16	36.00	65.00	60.94	60.66	0.46	37.00	37.28	0.75	39.00	39.00	0.01	653.00	657.32	0.66
17	45.00	60.00	67.20	66.70	0.75	24.00	24.12	0.51	21.00	25.34	20.64	690.00	430.50	37.61
18	30.00	60.00	74.40	74.92	0.70	31.00	30.86	0.44	24.00	24.77	3.20	588.00	563.93	4.09
19	45.00	85.00	55.25	55.44	0.34	27.00	27.03	0.11	34.00	34.92	2.71	372.00	364.10	2.12
20	31.00	50.00	85.00	85.18	0.21	42.00	41.75	0.60	38.00	39.77	4.67	834.00	777.67	6.75
21	25.00	100.00	57.14	57.19	0.08	42.00	41.96	0.10	34.00	33.97	0.08	842.00	842.00	0.00
22	36.00	70.00	77.00	76.96	0.05	31.00	30.95	0.15	39.00	38.95	0.12	761.00	760.86	0.02
23	52.00	70.00	70.00	70.03	0.05	31.00	31.02	0.08	42.00	42.40	0.95	622.00	621.56	0.07
24	25.00	50.00	61.25	61.20	0.08	30.00	30.00	0.00	36.00	35.98	0.06	529.00	528.49	0.10
25	26.00	55.00	93.50	93.17	0.35	44.00	43.19	1.84	39.00	40.94	4.97	954.00	983.30	3.07

Table S5

6.3 – NIV Matching Results to Patient Data

Digital Twin ID (DT ID)	Resp. Rate (bpm)	FiO2 (%)	PaO2 (mm Hg)			pCO2 (mmHg)			dPes VS dPpl (cmH2O)			Tidal Volume (ml)		
			Data	Simulation	% Error	Data	Simulation	% Error	Data	Simulation	% Error	Data	Simulation	% Error
1	57.00	45.00	87.30	84.91	2.74	31.00	29.13	6.03	40.00	42.44	6.10	480.70	365.20	24.03
2	28.00	60.00	67.50	67.32	0.26	34.00	33.96	0.12	32.00	32.15	0.46	616.00	608.90	1.15
3	45.00	50.00	72.00	71.95	0.07	31.00	30.93	0.23	49.00	49.03	0.06	840.00	821.20	2.24
4	34.00	45.00	76.50	76.49	0.01	39.80	39.73	0.18	42.00	42.04	0.10	751.00	747.60	0.45
5	50.00	45.00	60.30	60.19	0.18	31.00	30.86	0.45	27.00	27.82	3.04	452.00	430.80	4.69
6	46.00	55.00	78.57	79.59	1.29	25.00	24.06	3.76	38.00	37.85	0.39	697.00	572.00	17.93
7	34.00	60.00	60.00	59.98	0.03	36.00	35.92	0.22	38.00	38.41	1.08	882.00	867.90	1.60
8	42.00	60.00	76.00	75.96	0.05	29.00	28.71	0.99	42.00	43.56	3.72	875.00	819.96	6.29
9	42.00	85.00	58.40	59.52	1.91	35.00	34.40	1.72	28.00	32.60	16.41	786.00	591.75	24.71
10	20.00	55.00	73.70	73.87	0.23	44.00	43.97	0.06	21.00	21.62	2.94	656.00	634.59	3.26
11	28.00	50.00	66.25	66.17	0.12	42.00	41.77	0.55	31.00	31.15	0.48	800.00	790.63	1.17
12	26.00	70.00	70.00	69.59	0.58	19.00	18.71	1.50	38.00	38.21	0.55	738.00	700.06	5.14
13	25.00	40.00	90.00	89.91	0.10	34.00	33.95	0.14	32.00	32.05	0.17	1068.00	1060.40	0.71
14	26.00	70.00	93.33	92.97	0.39	44.00	41.25	6.25	22.00	23.58	7.18	672.00	599.30	10.82
15	38.00	60.00	60.00	59.96	0.07	42.00	42.01	0.02	42.00	41.97	0.07	852.00	851.47	0.06
16	36.00	65.00	60.94	60.66	0.46	37.00	37.28	0.75	39.00	39.00	0.01	653.00	657.32	0.66
17	45.00	60.00	67.20	66.70	0.75	24.00	24.12	0.51	21.00	25.34	20.64	690.00	430.50	37.61
18	30.00	60.00	74.40	74.92	0.70	31.00	30.86	0.44	24.00	24.77	3.20	588.00	563.93	4.09
19	45.00	85.00	55.25	55.44	0.34	27.00	27.03	0.11	34.00	34.92	2.71	372.00	364.10	2.12
20	31.00	50.00	85.00	85.18	0.21	42.00	41.75	0.60	38.00	39.77	4.67	834.00	777.67	6.75
21	25.00	100.00	57.14	57.19	0.08	42.00	41.96	0.10	34.00	33.97	0.08	842.00	842.00	0.00
22	36.00	70.00	77.00	76.96	0.05	31.00	30.95	0.15	39.00	38.95	0.12	761.00	760.86	0.02
23	52.00	70.00	70.00	70.03	0.05	31.00	31.02	0.08	42.00	42.40	0.95	622.00	621.56	0.07
24	25.00	50.00	61.25	61.20	0.08	30.00	30.00	0.00	36.00	35.98	0.06	529.00	528.49	0.10
25	26.00	55.00	93.50	93.17	0.35	44.00	43.19	1.84	39.00	40.94	4.97	954.00	983.30	3.07

Table S6

Section 7 – Digital Twin Output Tables

7.1 – HFNC Results

7.1.1 – HFNC Respiratory Effort

**DT ID**	**1**	**2**	**3**	**4**	**5**	**6**	**7**	**8**	**9**	**10**	**11**	**12**	**13**	**14**	**15**	**16**	**17**	**18**	**19**	**20**	**21**	**22**	**23**	**24**	**25**
**Respiratory Rate (bpm)**	57.0	28.0	45.0	34.0	50.0	46.0	34.0	42.0	42.0	20.0	28.0	26.0	25.0	26.0	38.0	36.0	45	30.0	45.0	31.0	25.0	36.0	52.0	25.0	26.0
**Muscle Pressure (cmH_2O)**	-30.0	-29.7	-41.1	-43.0	-21.7	-30.0	-44.9	-38.3	-25.2	-24.4	-31.4	-31.4	-37.6	-20.3	-44.1	-37.4	-15.9	-23.6	-26.6	-38.2	-34.6	-34.0	-31.1	-29.0	-40.8
**HFNC Generated PEEP (cmH_2O)**	11.2	11.9	10.0	12.0	8.3	9.5	9.3	8.8	8.7	11.3	9.6	12.3	11.7	10.1	10.4	9.8	9.6	10.4	12.1	10.5	10.9	10.8	12.1	16.6	11.3
**Total Inspiratory Pressure (cmH_2O)**	41.2	41.6	51.1	55.0	30.0	39.5	54.2	47.1	33.9	35.7	41.0	43.7	49.3	30.4	54.5	47.2	25.5	34.0	38.7	48.7	45.5	44.8	43.2	45.6	52.1
**Tidal Volume (ml)**	365.2	608.9	821.2	747.6	430.8	572.0	867.9	820.0	591.8	634.6	790.6	700.1	1060.4	599.3	851.5	766.6	287.3	555.1	364.1	777.7	1011.1	709.4	509.0	528.5	983.3

Table S7

7.1.2 – HFNC Blood-Gases

DT ID	1	2	3	4	5	6	7	8	9	10	11	12	13	14	15	16	17	18	19	20	21	22	23	24	25
PaO2 (mm Hg)	84.9	67.3	71.9	76.5	60.2	79.6	60.0	76.0	59.5	73.9	66.2	69.6	89.9	93.0	60.0	60.9	67.9	74.3	55.4	85.2	56.5	76.1	67.9	61.2	93.2
SaO2 (%)	96.9	93.2	94.7	94.1	91.4	97.0	89.9	95.7	90.2	92.6	90.9	96.7	96.9	96.4	88.1	90.2	95.6	95.3	91.1	95.4	86.4	95.6	93.6	92.1	96.2
PaCO2 (mm Hg)	29.1	34.0	30.9	39.7	30.9	24.1	35.9	28.7	34.4	44.0	41.8	18.7	34.0	41.3	42.0	36.9	23.8	29.9	27.0	41.7	41.5	30.9	30.9	30.0	43.2
pH	7.4	7.4	7.4	7.3	7.4	7.5	7.4	7.4	7.4	7.3	7.3	7.6	7.4	7.3	7.3	7.4	7.5	7.4	7.5	7.3	7.3	7.4	7.4	7.4	7.3
PF (mm Hg)	188.7	112.2	143.9	170.0	133.7	144.7	100.0	126.6	70.0	134.3	132.3	99.4	224.8	132.8	99.9	93.6	113.1	123.8	65.2	170.4	56.5	108.7	96.9	122.4	169.4

Table S8

7.1.3 – HFNC Respiratory Mechanics

DT ID	1	2	3	4	5	6	7	8	9	10	11	12	13	14	15	16	17	18	19	20	21	22	23	24	25
Resp. Sys. Compliance ($ml\ cmH_2O^{-1}$)	12.1	26.3	23.9	25.5	21.9	21.0	30.1	25.3	25.5	40.6	34.2	27.0	44.0	36.1	23.2	25.1	19.8	29.4	14.9	26.1	40.1	24.5	16.2	23.0	32.9
Lung Compliance ($ml\ cmH_2O^{-1}$)	13.7	32.7	31.0	33.1	27.4	27.0	37.0	32.4	32.0	55.7	44.4	35.1	63.1	48.6	27.9	34.0	23.7	40.8	17.2	32.1	55.7	29.6	19.9	27.2	42.6
Chest Wall Compliance ($ml\ cmH_2O^{-1}$)	108.4	134.4	104.6	110.3	107.6	95.4	162.1	115.7	125.5	150.5	148.9	117.3	145.8	140.9	139.1	96.8	119.5	105.5	113.1	139.3	143.3	140.2	88.4	147.3	144.0
End Exp. Lung Volume (ml)	3201.4	3933.9	3079.1	3330.1	3122.4	2639.1	3638.3	3711.6	3628.5	3996.0	3913.5	3376.1	4223.0	3894.3	3490.3	3161.6	3456.1	3429.3	2878.4	3708.4	3842.0	4205.4	2715.1	3760.9	4000.1
Baseline End Exp. Lung Volume (ml)	2051.6	3022.5	1944.9	2382.7	2498.4	1761.3	2887.1	2877.9	2765.2	2720.3	2906.0	2462.5	3165.3	2854.9	2374.0	2471.3	2725.0	2312.6	2240.4	2789.7	2823.5	3187.5	1854.8	2346.9	3043.4
ΔPleural Pressure (cmH_2O)	42.4	32.1	49.0	42.0	27.8	37.8	38.4	43.6	32.6	21.6	31.1	38.2	32.1	23.6	42.0	39.4	21.7	26.6	34.9	39.8	35.3	41.2	45.0	36.0	40.9
ΔTranspul. Pressure (cmH_2O)	26.7	18.6	26.5	22.6	15.7	21.2	23.5	25.3	18.5	11.4	17.8	19.9	16.8	12.3	30.6	22.6	12.1	13.6	21.2	24.2	18.2	23.9	25.6	19.4	23.1

Table S9

7.1.4 – HFNC Lung Injury Indices

DT ID	1	2	3	4	5	6	7	8	9	10	11	12	13	14	15	16	17	18	19	20	21	22	23	24	25
**Dynamic Lung Strain**	0.18	0.20	0.42	0.31	0.17	0.32	0.30	0.28	0.21	0.23	0.27	0.32	0.24	0.21	0.36	0.31	0.11	0.24	0.16	0.28	0.36	0.22	0.27	0.23	0.32
**Static Lung Strain**	0.56	0.30	0.58	0.40	0.25	0.50	0.26	0.29	0.31	0.47	0.35	0.55	0.33	0.36	0.47	0.28	0.27	0.48	0.28	0.33	0.36	0.32	0.46	0.60	0.31
**Total Lung Strain**	0.74	0.50	1.01	0.71	0.42	0.82	0.56	0.57	0.53	0.70	0.62	0.86	0.57	0.57	0.83	0.59	0.37	0.72	0.45	0.61	0.72	0.54	0.74	0.83	0.64
**Total Lung Stress (cmH_2O)**	26.7	18.6	26.5	22.6	15.7	21.2	23.5	25.3	18.5	11.4	17.8	19.9	16.8	12.3	30.6	22.6	12.1	13.6	21.2	24.2	18.2	23.9	25.6	19.4	23.1
**Driving Pressure (cmH_2O)**	13.7	13.1	19.8	19.6	10.7	15.0	17.0	16.3	13.1	9.0	12.9	15.3	15.9	9.6	21.5	15.5	8.6	14.5	14.0	16.4	18.0	16.6	17.5	17.4	17.3
**Mech. Power ($J\ min^{-1}$)**	27.3	15.6	48.0	28.1	16.6	27.4	33.9	42.7	22.5	7.1	19.3	17.8	21.9	9.4	48.5	30.5	7.7	11.1	17.0	28.6	22.5	30.0	33.2	12.6	28.9

Table S10

7.2 – NIV Results

7.2.1 – NIV Respiratory Effort

DT ID	1	2	3	4	5	6	7	8	9	10	11	12	13	14	15	16	17	18	19	20	21	22	23	24	25
Respiratory Rate (bpm)	37.0	24.0	32.0	31.0	47.0	30.0	37.0	38.0	30.0	18.0	23.0	26.0	24.0	40.0	38.0	28.0	42	26.0	30.0	31.0	36.0	28.0	38.0	25.0	24.0
Muscle Pressure (cmH_2O)	-16.1	-31.3	-14.4	-40.5	-13.3	-22.6	-32.0	-54.9	-18.8	-15.6	-9.3	-51.0	-37.5	-19.3	-38.5	-13.0	-20.0	-25.0	-20.2	-42.2	-15.0	-29.3	-40.9	-38.3	-40.1
Insp. Pressure Support (cmH_2O)	18.0	2.0	26.0	6.0	12.0	12.0	4.0	10.0	36.0	22.0	22.0	17.0	20.0	36.0	7.0	20.0	20.0	22.0	24.0	4.0	28.0	14.0	4.0	9.0	22.0
PEEP (cmH_2O)	10.0	8.0	6.0	10.0	6.0	8.0	8.0	6.0	10.0	8.0	5.0	8.0	8.0	8.0	10.0	8.0	6.0	6.0	6.0	8.0	10.0	11.0	8.0	8.0	10.0
Total Inspiratory Pressure (cmH_2O)	44.1	41.3	46.4	56.5	31.3	42.6	44.0	70.9	64.8	45.6	36.3	76.0	65.5	63.3	55.5	41.0	46.0	53.0	50.2	54.2	53.0	54.3	52.9	55.3	72.1
Tidal Volume (ml)	418.9	785.9	569.8	906.9	313.9	555.5	730.5	839.0	687.9	655.5	586.5	810.5	877.4	607.9	818.4	558.7	534.0	870.1	713.2	747.7	582.9	1015.9	622.0	752.9	962.8

Table S11

7.2.2 – NIV Blood Gases

DT ID	1	2	3	4	5	6	7	8	9	10	11	12	13	14	15	16	17	18	19	20	21	22	23	24	25
PaO2 (mm Hg)	59.3	67.0	71.5	68.9	61.1	62.0	63.2	61.7	60.0	61.9	69.4	57.7	87.8	74.9	81.2	66.2	64.1	59.9	63.2	60.5	70.8	60.0	68.0	68.0	66.0
SaO2 (%)	89.3	93.6	94.6	93.2	91.3	93.2	91.1	92.3	89.8	88.3	91.3	93.8	96.6	95.1	94.7	90.9	95.1	93.2	94.7	89.1	94.2	91.5	92.8	94.3	91.0
PaCO2 (mm Hg)	37.3	34.7	32.9	35.8	31.8	32.4	36.3	30.8	36.0	44.8	43.7	20.7	33.9	31.9	41.0	41.9	22.7	26.7	26.0	39.7	32.0	30.0	35.0	28.9	42.3
pH	7.4	7.4	7.4	7.4	7.4	7.5	7.4	7.4	7.4	7.3	7.3	7.6	7.4	7.4	7.3	7.3	7.6	7.5	7.6	7.3	7.4	7.4	7.4	7.4	7.3
PF (mm Hg)	118.7	134.0	158.9	125.4	111.0	88.5	97.2	102.8	99.9	137.6	154.2	96.1	159.7	149.9	180.5	147.1	128.2	85.6	140.4	86.4	118.0	100.0	151.1	169.9	110.0

Table S12

7.2.3 – NIV Respiratory Mechanics

DT ID	1	2	3	4	5	6	7	8	9	10	11	12	13	14	15	16	17	18	19	20	21	22	23	24	25
**Resp. Sys. Compliance** $(ml\ cmH_2O^{-1})$	35.1	28.7	51.4	32.8	32.4	38.0	27.4	22.7	58.2	77.8	101.5	28.3	39.9	40.1	27.6	64.8	34.9	57.2	45.3	26.6	49.6	54.0	17.5	33.5	36.8
**Lung Compliance** $(ml\ cmH_2O^{-1})$	52.0	36.6	101.0	46.6	46.3	63.1	32.9	28.3	108.5	161.1	318.7	37.3	54.9	56.1	34.4	196.2	49.2	124.9	75.5	32.9	76.0	87.7	21.8	43.3	49.4
**Chest Wall Compliance** $(ml\ cmH_2O^{-1})$	108.4	134.4	104.6	110.3	107.6	95.4	162.1	115.7	125.5	150.5	148.9	117.3	145.8	140.9	139.1	96.8	119.5	105.5	113.1	139.3	143.3	140.2	88.4	147.3	144.0
**End Exp. Lung Volume (ml)**	3286.8	4236.3	3529.5	3647.5	3535.5	2897.9	3647.2	3474.1	3679.4	4399.8	4385.2	3437.8	4653.7	4126.4	3934.7	3632.9	3672.5	3849.5	3304.1	3789.6	4160.8	4263.8	3039.4	4251.2	3602.3
**Baseline End Exp. Lung Volume (ml)**	2277.2	3111.9	2445.1	2392.8	2455.0	2372.9	2778.7	1799.5	2442.9	3526.7	3352.0	2352.0	3369.9	2786.8	2172.0	2597.7	2435.6	2544.1	2416.5	3168.4	2983.4	2767.8	2231.2	2250.8	2743.0
**ΔPleural Pressure** (cmH_2O)	11.2	40.0	10.3	32.7	9.8	15.0	36.8	35.9	11.0	8.0	5.1	31.1	23.8	15.5	32.4	8.6	12.2	14.0	16.3	36.4	11.8	19.0	38.0	30.9	29.0
**ΔTranspul. Pressure** (cmH_2O)	8.1	21.5	5.6	19.5	6.8	8.8	22.2	29.7	6.3	4.1	1.8	21.7	16.0	10.8	23.8	2.8	10.9	7.0	9.4	22.7	7.7	11.6	28.5	17.4	19.5
**Intrinsic PEEP** (cmH_2O)	1.3	3.6	2.7	3.2	2.9	1.5	3.0	4.1	2.4	1.5	1.6	3.3	3.1	3.7	4.4	1.5	2.8	2.1	3.2	2.7	3.2	2.4	3.2	4.6	2.1

Table S13

7.2.4 – NIV Lung Injury Indices

DT ID	1	2	3	4	5	6	7	8	9	10	11	12	13	14	15	16	17	18	19	20	21	22	23	24	25
Dynamic Lung Strain	0.18	0.25	0.23	0.38	0.13	0.23	0.26	0.47	0.28	0.19	0.17	0.34	0.26	0.22	0.38	0.22	0.22	0.34	0.30	0.24	0.20	0.37	0.28	0.33	0.35
Static Lung Strain	0.44	0.36	0.44	0.52	0.44	0.22	0.31	0.93	0.51	0.25	0.31	0.46	0.38	0.48	0.81	0.40	0.51	0.51	0.37	0.20	0.39	0.54	0.36	0.89	0.31
Total Lung Strain	0.63	0.61	0.68	0.90	0.57	0.46	0.58	1.40	0.79	0.43	0.48	0.81	0.64	0.70	1.19	0.61	0.73	0.86	0.66	0.43	0.59	0.91	0.64	1.22	0.66
Total Lung Stress (cmH_2O)	8.1	21.5	5.6	19.5	6.8	8.8	22.2	29.7	6.3	4.1	1.8	21.7	16.0	10.8	23.8	2.8	10.9	7.0	9.4	22.7	7.7	11.6	28.5	17.4	19.5
Driving Pressure (cmH_2O)	19.3	19.9	21.5	17.3	12.6	13.6	16.7	18.2	36.5	22.9	22.9	19.8	16.1	30.1	18.9	20.9	22.2	18.2	25.7	15.9	29.8	16.9	18.3	18.3	24.8
Mech. Power ($J\ min^{-1}$)	6.1	19.9	5.0	26.8	4.9	7.2	29.4	46.4	6.4	2.4	1.2	22.4	16.5	12.9	36.3	2.2	11.9	7.7	9.9	25.8	7.9	16.1	33.0	16.0	22.1

Table S14

Section 8 – Spontaneous Ventilation Contribution Isolation

To separate out the effects of the spontaneous respiratory effort and the additional support being provided, the digital twins had their respiratory support removed while their levels of respiratory effort were maintained constant. Reported below is the contribution of the spontaneous respiratory effort to the lung injury indices.

8.1 – HFNC

DT ID	1	2	3	4	5	6	7	8	9	10	11	12	13	14	15	16	17	18	19	20	21	22	23	24	25
**Total Lung Strain**	0.26	0.17	0.56	0.33	0.19	0.51	0.22	0.36	0.26	0.10	0.25	-0.13	0.32	0.19	0.40	0.13	0.13	0.40	-0.01	0.35	0.27	0.28	0.54	0.01	0.12
**Total Lung Stress (cmH_2O)**	26.6	19.5	28.8	23.2	16.1	24.6	20.9	26.0	19.3	11.6	19.1	20.6	17.2	15.2	32.2	24.4	16.1	15.9	18.4	24.9	21.6	23.1	28.4	18.9	22.2
**Driving Pressure (cmH_2O)**	16.6	13.8	19.9	19.1	12.1	10.7	16.7	16.8	14.6	7.2	12.7	16.2	13.2	12.1	20.6	12.4	10.9	10.9	12.0	16.1	13.3	13.4	16.9	12.9	16.7
**Mech. Power ($J\ min^{-1}$)**	17.6	11.2	36.3	19.7	13.9	24.6	17.9	38.0	19.7	3.7	15.3	7.0	17.3	7.7	39.5	22.8	13.2	9.7	10.3	26.3	16.3	23.8	39.1	5.5	14.6

Table S15

8.2 – NIV

DT ID	1	2	3	4	5	6	7	8	9	10	11	12	13	14	15	16	17	18	19	20	21	22	23	24	25
**Total Lung Strain**	0.10	0.23	0.07	0.35	0.02	0.15	0.24	0.59	0.11	0.07	-0.01	0.27	0.13	0.05	0.30	0.06	0.08	0.15	0.11	0.22	0.04	0.21	0.25	0.14	0.26
**Total Lung Stress (cmH_2O)**	10.2	24.5	10.4	23.5	11.1	14.5	24.4	36.1	13.0	8.2	4.9	31.5	22.1	15.8	27.4	8.0	14.2	13.4	14.9	24.9	11.7	18.5	30.6	21.5	26.8
**Driving Pressure (cmH_2O)**	4.8	19.1	7.6	16.5	7.4	9.6	17.9	17.0	9.5	6.3	4.1	17.5	12.0	9.9	17.6	5.8	6.9	8.5	11.2	15.4	7.4	10.8	19.4	12.2	14.8
**Mech. Power ($J\ min^{-1}$)**	3.7	15.2	3.1	21.3	2.4	6.4	21.4	36.4	4.4	2.4	1.0	20.9	11.5	5.8	21.4	2.4	5.8	6.3	5.3	20.5	3.4	12.4	22.2	9.4	19.3

Table S16

Section 9 – NIV Support Setting

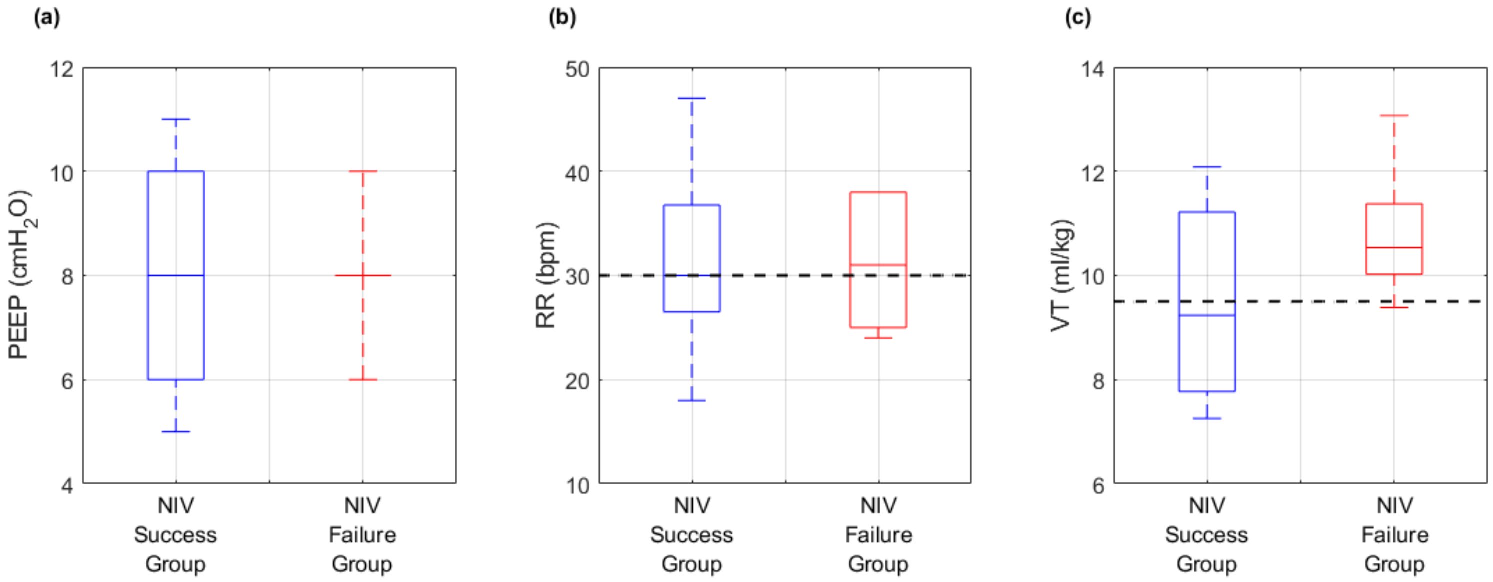

Figure S4 – (a) The PEEP provided to the success (blue) and failure (red) groups during NIV. (b-c) The respiratory rate and tidal volume recorded from patients used to titrate the level of inspiratory pressure support (PS) provided. The black dashed line indicates the targets. All three panels present he median(s), interquartile range(s), and full range(s).

Section 10 – Tidal Recruitment

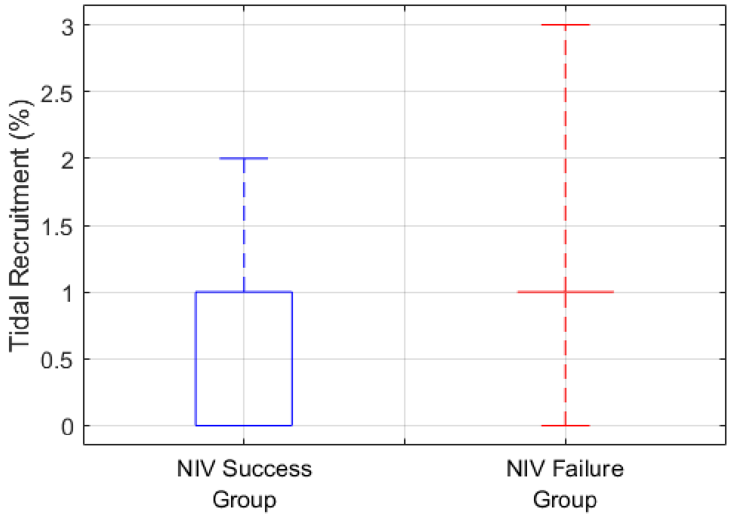

Figure S5 – The % of tidal recruitment observed for the success (blue) and failure (red) groups during NIV. Please note that he median for the success case is 1%, which coincides with the upper bound of the interquartile range, hence why it is not easily visible.

References

[1] J. G. Hardman, N. M. Bedforth, A. B. Ahmed, R. P. Mahajan, and A. R. Aitkenhead, “A physiology simulator: validation of its respiratory components and its ability to predict the patient’s response to changes in mechanical ventilation,” *Br J Anaesth*, vol. 81, no. 3, pp. 327–332, 1998, doi: 10.1093/BJA/81.3.327.

[2] J. G. Hardman and N. M. Bedforth, “Estimating venous admixture using a physiological simulator,” *Br J Anaesth*, vol. 82, no. 3, pp. 346–349, 1999, doi: 10.1093/BJA/82.3.346.

[3] J. G. Hardman and A. R. Aitkenhead, “Estimation of alveolar deadspace fraction using arterial and end-tidal CP2: A factor analysis using a physiological simulation,” *Anaesth Intensive Care*, vol. 27, no. 5, pp. 452–458, Jan. 1999, doi: 10.1177/0310057x9902700503.

[4] J. G. Hardman and A. R. Aitkenhead, “Validation of an Original Mathematical Model of CO2 Elimination and Dead Space Ventilation,” *Anesth Analg*, vol. 97, no. 6, pp. 1840–1845, 2003, doi: 10.1213/01.ANE.0000090315.45491.72.

[5] J. G. Hardman and J. S. Wills, “The development of hypoxaemia during apnoea in children: a computational modelling investigation,” *Br J Anaesth*, vol. 97, no. 4, pp. 564–570, Oct. 2006, doi: 10.1093/BJA/AEL178.

[6] A. Das, Z. Gao, P. P. Menon, J. G. Hardman, and D. G. Bates, “A systems engineering approach to validation of a pulmonary physiology simulator for clinical applications,” *J R Soc Interface*, vol. 8, no. 54, pp. 44–55, Jan. 2011, doi: 10.1098/RSIF.2010.0224.

[7] R. A. McCahon, M. O. Columb, R. P. Mahajan, and J. G. Hardman, “Validation and application of a high-fidelity, computational model of acute respiratory distress syndrome to the examination of the indices of oxygenation at constant lung-state,” *Br J Anaesth*, vol. 101, no. 3, pp. 358–365, 2008, doi: 10.1093/BJA/AEN181.

[8] P. M. Hassoun *et al.*, “Prediction of Arterial Oxygen Tension: Validation of a Novel Formula,” *https://doi.org/10.1164/ajrccm.182.3.435*, vol. 182, no. 3, pp. 435–436, Dec. 2012, doi: 10.1164/AJRCCM.182.3.435.

[9] L. Weaver *et al.*, “High risk of patient self-inflicted lung injury in COVID-19 with frequently encountered spontaneous breathing patterns: a computational modelling study,” *Annals of Intensive Care 2021 11:1*, vol. 11, no. 1, pp. 1–8, Jul. 2021, doi: 10.1186/S13613-021-00904-7.

[10] L. Weaver *et al.*, “Optimising respiratory support for early COVID-19 pneumonia: a computational modelling study,” *Br J Anaesth*, vol. 128, no. 6, pp. 1052–1058, 2022, doi: 10.1016/j.bja.2022.02.037.

[11] B. E. Marshall, W. R. Clarke, A. T. Costarino, L. Chen, F. Miller, and C. Marshall, “The dose-response relationship for hypoxic pulmonary vasoconstriction,” *Respir Physiol*, vol. 96, no. 2–3, pp. 231–247, May 1994, doi: 10.1016/0034-5687(94)90129-5.

[12] J. W. Severinghaus, “Simple, accurate equations for human blood O2 dissociation computations,” *J Appl Physiol Respir Environ Exerc Physiol*, vol. 46, no. 3, pp. 599–602, 1979, doi: 10.1152/JAPPL.1979.46.3.599.

[13] J. W. Severinghaus, “Blood gas calculator,” *J Appl Physiol*, vol. 21, no. 3, pp. 1108–1116, 1966, doi: 10.1152/JAPPL.1966.21.3.1108.

[14] A. R. Douglas, N. L. Jones, and J. W. Reed, “Calculation of whole blood CO2 content,” *https://doi.org/10.1152/jappl.1988.65.1.473*, vol. 65, no. 1, pp. 473–477, 1988, doi: 10.1152/JAPPL.1988.65.1.473.

[15] G. J. McHardy, “The relationship between the differences in pressure and content of carbon dioxide in arterial and venous blood.,” *Clin Sci*, vol. 32, no. 2, pp. 299–309, Apr. 1967.

[16] G. R. Kelman and J. F. Nunn, “Nomograms for correction of blood Po2, Pco2, pH, and base excess for time and temperature.,” *J Appl Physiol*, vol. 21, no. 5, pp. 1484–1490, Sep. 1966, doi: 10.1152/jappl.1966.21.5.1484.

[17] O. Siggaard-Andersen, “The van Slyke equation.,” *Scand J Clin Lab Invest Suppl*, vol. 146, pp. 15–20, 1977, doi: 10.3109/00365517709098927.

[18] B. Lachmann, “Open up the lung and keep the lung open,” *Intensive Care Med*, vol. 18, no. 6, pp. 319–321, Jun. 1992, doi: 10.1007/BF01694358.

[19] K. G. Hickling, “The pressure-volume curve is greatly modified by recruitment. A mathematical model of ARDS lungs,” *Am J Respir Crit Care Med*, vol. 158, no. 1, pp. 194–202, 1998, doi: 10.1164/AJRCCM.158.1.9708049.

[20] J. H. T. Bates and C. G. Irvin, “Time dependence of recruitment and derecruitment in the lung: a theoretical model,” *J Appl Physiol (1985)*, vol. 93, no. 2, pp. 705–713, 2002, doi: 10.1152/JAPPLPHYSIOL.01274.2001.

[21] J. S. Mecklenburgh and W. W. Mapleson, “Ventilatory assistance and respiratory muscle activity. 2: Simulation with an adaptive active (‘aa’ or ‘a-squared’) model lung,” *Br J Anaesth*, vol. 80, no. 4, pp. 434–439, 1998, doi: 10.1093/BJA/80.4.434.

[22] A. Albanese, L. Cheng, M. Ursino, and N. W. Chbat, “An integrated mathematical model of the human cardiopulmonary system: model development,” *American Journal of Physiology-Heart and Circulatory Physiology*, vol. 310, no. 7, pp. H899–H921, Apr. 2016, doi: 10.1152/ajpheart.00230.2014.

[23] E. Agostoni and J. Mead, *Statics of the respiratory system*, vol. 1. American Physiological Soc, 1964.

[24] R. Lampe, T. Blumenstein, V. Turova, and A. Alves-Pinto, “Lung vital capacity and oxygen saturation in adults with cerebral palsy,” *Patient Prefer Adherence*, vol. 8, p. 1691, 2014, doi: 10.2147/PPA.S72575.

[25] J. Stocks and P. H. Quanjer, “REFERENCE VALUES FOR RESIDUAL VOLUME, FUNCTIONAL RESIDUAL CAPACITY AND TOTAL LUNG CAPACITY,” *European Respiratory Journal*, pp. 492–506, 1995, doi: 10.1183/09031936.95.08030492.

[26] A. Protti, E. Votta, and L. Gattinoni, “Which is the most important strain in the pathogenesis of ventilator-induced lung injury: dynamic or static?,” *Curr Opin Crit Care*, vol. 20, no. 1, pp. 33–38, Feb. 2014, doi: 10.1097/MCC.0000000000000047.

[27] M. Eikermann and M. F. V. Melo, “Therapeutic range of spontaneous breathing during mechanical ventilation,” *Anesthesiology*, vol. 120, no. 3, p. 536, 2014, doi: 10.1097/ALN.0000000000000126.

[28] M. Cressoni *et al.*, “Mechanical Power and Development of Ventilator-induced Lung Injury,” *Anesthesiology*, vol. 124, no. 5, pp. 1100–1108, May 2016, doi: 10.1097/ALN.0000000000001056.

[29] G. Bellani, A. Grassi, S. Sosio, and G. Foti, “Plateau and driving pressure in the presence of spontaneous breathing,” *Intensive Care Med*, vol. 45, no. 1, pp. 97–98, 2019, doi: 10.1007/S00134-018-5311-9.

[30] A. Protti *et al.*, “Lung stress and strain during mechanical ventilation: any safe threshold?,” *Am J Respir Crit Care Med*, vol. 183, no. 10, pp. 1354–1362, May 2011, doi: 10.1164/RCCM.201010-1757OC.

[31] D. Chiumello *et al.*, “Lung stress and strain during mechanical ventilation for acute respiratory distress syndrome,” *Am J Respir Crit Care Med*, vol. 178, no. 4, pp. 346–355, Aug. 2008, doi: 10.1164/RCCM.200710-1589OC.

[32] A. K. Baldomero, P. K. Skarda, and J. J. Marini, “Driving Pressure: Defining the Range,” *Respir Care*, vol. 64, no. 8, pp. 883–889, Aug. 2019, doi: 10.4187/RESPCARE.06599.

[33] F. Romitti *et al.*, “Mechanical power thresholds during mechanical ventilation: An experimental study,” *Physiol Rep*, vol. 10, no. 6, p. 15225, Mar. 2022, doi: 10.14814/PHY2.15225.

[34] R. Tonelli *et al.*, “Early Inspiratory Effort Assessment by Esophageal Manometry Predicts Noninvasive Ventilation Outcome in De Novo Respiratory Failure. A Pilot Study,” *Am J Respir Crit Care Med*, vol. 202, no. 4, pp. 558–567, Aug. 2020, doi: 10.1164/RCCM.201912-2512OC.
